# Coassembly of hypoxia-sensitive macrocyclic amphiphiles and extracellular vesicles for targeted kidney injury imaging and therapy

**DOI:** 10.1186/s12951-021-01192-w

**Published:** 2021-12-27

**Authors:** Yuan-Qiu Cheng, Yu-Xin Yue, Hong-Mei Cao, Wen-Chao Geng, Lan-Xing Wang, Xin-Yue Hu, Hua-Bin Li, Qiang Bian, Xiang-Lei Kong, Jian-Feng Liu, De-Ling Kong, Dong-Sheng Guo, Yue-Bing Wang

**Affiliations:** 1 Nankai University School of Medicine, Tianjin, 300071 China; 2 College of Chemistry, Key Laboratory of Functional Polymer Materials (Ministry of Education), State Key Laboratory of Elemento-Organic Chemistry, Nankai University, Tianjin, 300071 China; 3 Tianjin First Central Hospital, School of Medicine, Nankai University, Tianjin, China; 4 The Key Laboratory of Bioactive Materials, Ministry of Education, College of Life Sciences, Nankai University, Tianjin, 300071 China; 5 College of Chemistry, State Key Laboratory of Elemento-Organic Chemistry, Nankai University, Tianjin, 300071 China; 6 National Pesticide Engineering Research Center, College of Chemistry, Nankai University, Tianjin, 300071 China; 7 Key Laboratory of Radiopharmacokinetics for Innovative Drugs, Chinese Academy of Medical Sciences and Institute of Radiation Medicine, Chinese Academy of Medical Sciences & Peking Union Medical College, Tianjin, 300192 China

**Keywords:** Supramolecular chemistry, Extracellular vesicles, Macrocyclic amphiphile, Kidney hypoxia, Coassembly

## Abstract

**Background:**

Hypoxia is a major contributor to global kidney diseases. Targeting hypoxia is a promising therapeutic option against both acute kidney injury and chronic kidney disease; however, an effective strategy that can achieve simultaneous targeted kidney hypoxia imaging and therapy has yet to be established. Herein, we fabricated a unique nano-sized hypoxia-sensitive coassembly (Pc/C5A@EVs) via molecular recognition and self-assembly, which is composed of the macrocyclic amphiphile C5A, the commercial dye sulfonated aluminum phthalocyanine (Pc) and mesenchymal stem cell-excreted extracellular vesicles (MSC-EVs).

**Results:**

In murine models of unilateral or bilateral ischemia/reperfusion injury*,* MSC-EVs protected the Pc/C5A complex from immune metabolism, prolonged the circulation time of the complex, and specifically led Pc/C5A to hypoxic kidneys via surface integrin receptor α_4_β_1_ and α_L_β_2_, where Pc/C5A released the near-infrared fluorescence of Pc and achieved enhanced hypoxia-sensitive imaging. Meanwhile, the coassembly significantly recovered kidney function by attenuating cell apoptosis, inhibiting the progression of renal fibrosis and reducing tubulointerstitial inflammation. Mechanistically, the Pc/C5A coassembly induced M1-to-M2 macrophage transition by inhibiting the HIF-1α expression in hypoxic renal tubular epithelial cells (TECs) and downstream NF-κB signaling pathway to exert their regenerative effects.

**Conclusion:**

This synergetic nanoscale coassembly with great translational potential provides a novel strategy for precise kidney hypoxia diagnosis and efficient kidney injury treatment. Furthermore, our strategy of coassembling exogenous macrocyclic receptors with endogenous cell-derived membranous structures may offer a functional platform to address multiple clinical needs.

**Graphical Abstract:**

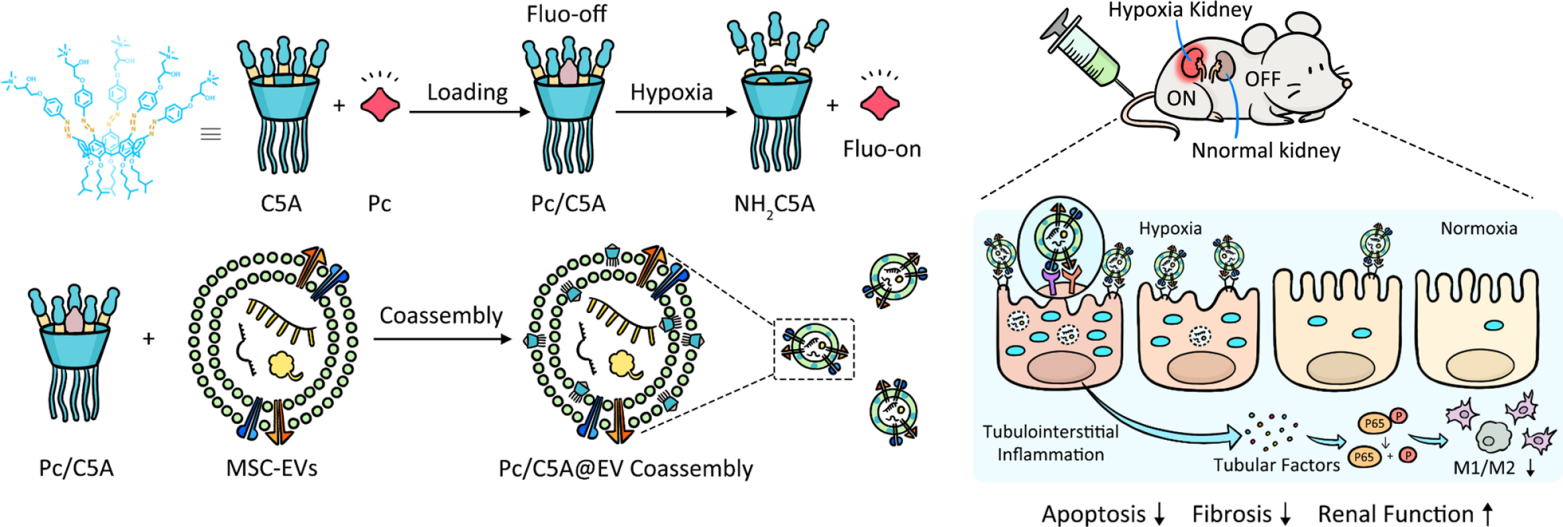

**Supplementary Information:**

The online version contains supplementary material available at 10.1186/s12951-021-01192-w.

## Background

Hypoxia is a critical mediator in kidney diseases worldwide, which plays a pivotal role in the initiation and progression of both acute kidney injury (AKI) and chronic kidney disease (CKD) [1, 2]. Due to the drastic reabsorption and excretion processes that occur in the renal tubules, especially in proximal tubular epithelial cells (TECs), the maximum oxygen demand of the kidneys is second only to the brain [3]; however, the unique renal vasculature architecture delivers a very limited oxygen supply to the renal tubules [3, 4]. Consequently, this prominent conflict between low supply and high demand makes TECs extremely vulnerable to hypoxic injury [5, 6]. In response to hypoxia, TECs convert to a pathologic secretory phenotype and produce multiple pro-inflammatory factors that recruit activated macrophages into the interstitium, thus triggering tubulointerstitial inflammation [6–8]. When hypoxia advances, the continuous activation of hypoxia-inducible factor-1 alpha (HIF-1α) in TECs stimulates partial epithelial-mesenchymal transition and facilitates the generation of tubulointerstitial fibrosis (TIF) [9, 10]. By persistently impairing peritubular capillaries and nephrons, hypoxia and fibrosis are exacerbated, eventually culminating in kidney failure [11]. Hypoxia promotes tubulointerstitial inflammation and TIF in the kidney; therefore, targeting hypoxia is a promising therapeutic option against a wide variety of kidney diseases. However, to target hypoxia, it is of paramount importance to first noninvasively and specifically image renal hypoxia [1]. Such imaging would help identify hypoxic areas, monitor treatment response, and potentially advance therapeutic strategies and effects [12]. To ultimately ameliorate renal hypoxia, a strategy that is able to fulfill simultaneous noninvasive imaging and targeted therapy is highly desirable but challenging.

Suitable tools for imaging renal hypoxia within tissues are presently lacking despite an increasing demand for the approaches to recognize and quantify hypoxic cells in the kidney [12]. Blood oxygen level-dependent magnetic resonance imaging (BOLD-MRI) is a strategy that is increasingly being investigated to monitor renal hypoxia [13, 14]. However, it is severely affected by hemoglobin concentration and thus is unable to precisely and reliably evaluate hypoxia [1, 4]. Because the degree of hypoxia is tightly associated with the local concentrations of azoreductase, a broad range of azobenzene (azo)-containing fluorescence probes have been fabricated based on the hypoxia-triggered molecular cleavage characteristics in a reductive microenvironment [15–19]. Most azo-reduction imaging approaches covalently conjugate a dye to an azo group [20, 21]. We recently developed a novel noncovalent fluorescence turn-on approach for imaging of hypoxia based on the molecular recognition between an azocalixarene and a dye [22]. We have applied this supramolecular strategy for tumor imaging and therapy [23, 24]. Our host–guest hypoxia imaging strategy is also amenable to tracing kidney hypoxia in vivo because our imaging approach exhibits a few intrinsic advantages, including the use of commercial probes released with high fidelity, much easier preparation than covalent synthesis, and easy adaptability to other probes or drugs to construct a universal platform. However, certain obstacles, including deficiency of specific targeting ability, and rapid clearance by the immune system, need to be properly addressed before our host–guest strategy can be used for kidney hypoxia imaging.

Cell membrane coating technology presents an adept approach that endows nanocarriers with characteristics of the source cells from which their membrane is derived, enabling a variety of functions and extra applications [25–27]. As endogenous membrane-bound nanoscale vesicles, extracellular vesicles (EVs) have attracted significant interest as fascinating drug delivery vectors for different diseases [28]. EVs potentially offer many benefits due to their natural complexity, such as immune escape and decreased off-target effects, thereby increasing their circulation time and cellular uptake [29, 30]. Another tetraspanin, CD9, facilitates endocytosis by target cells via direct membrane fusion, which evades lysosomal trapping [31, 32]. Very recently, our research demonstrated that mesenchymal stem cells derived EVs (MSC-EVs) have a specific-tropism capability to ischemic kidneys [33], which will inevitably increase their delivery efficiency. In addition to their vehicle property, MSC-EVs can directly exert regenerative effects to kidney disease by grafting the native biological functions of their parental MSCs [34, 35]. However, details about MSC-EV-mediated signaling pathways in renal hypoxia, such as downstream adaptor proteins or phosphorylation steps, remain largely unknown. Of note, MSC-EVs also hold excellent translational potential. MSCs are compatible with commercially sustainable production of EVs [28, 36], and some clinical trials of MSC-EVs in kidney disease patients are being conducted (clinicaltrials.gov) [37]. Consequently, integrating MSC-EVs and azocalixarenes into one ensemble may create an innovative coassembly to address the formidable challenges of specific homing, long circulation, hypoxia turn-on imaging and targeted therapy, thereby leading to the recovery of injured kidneys. To the best of our knowledge, a coassembly based on macrocyclic amphiphiles and EVs for simultaneous hypoxia-sensitive imaging and injury treatment has yet to be reported. This system holds great potential for future treatment of kidney diseases, and may bring exciting possibilities to other disease therapies.

**Scheme 1:**
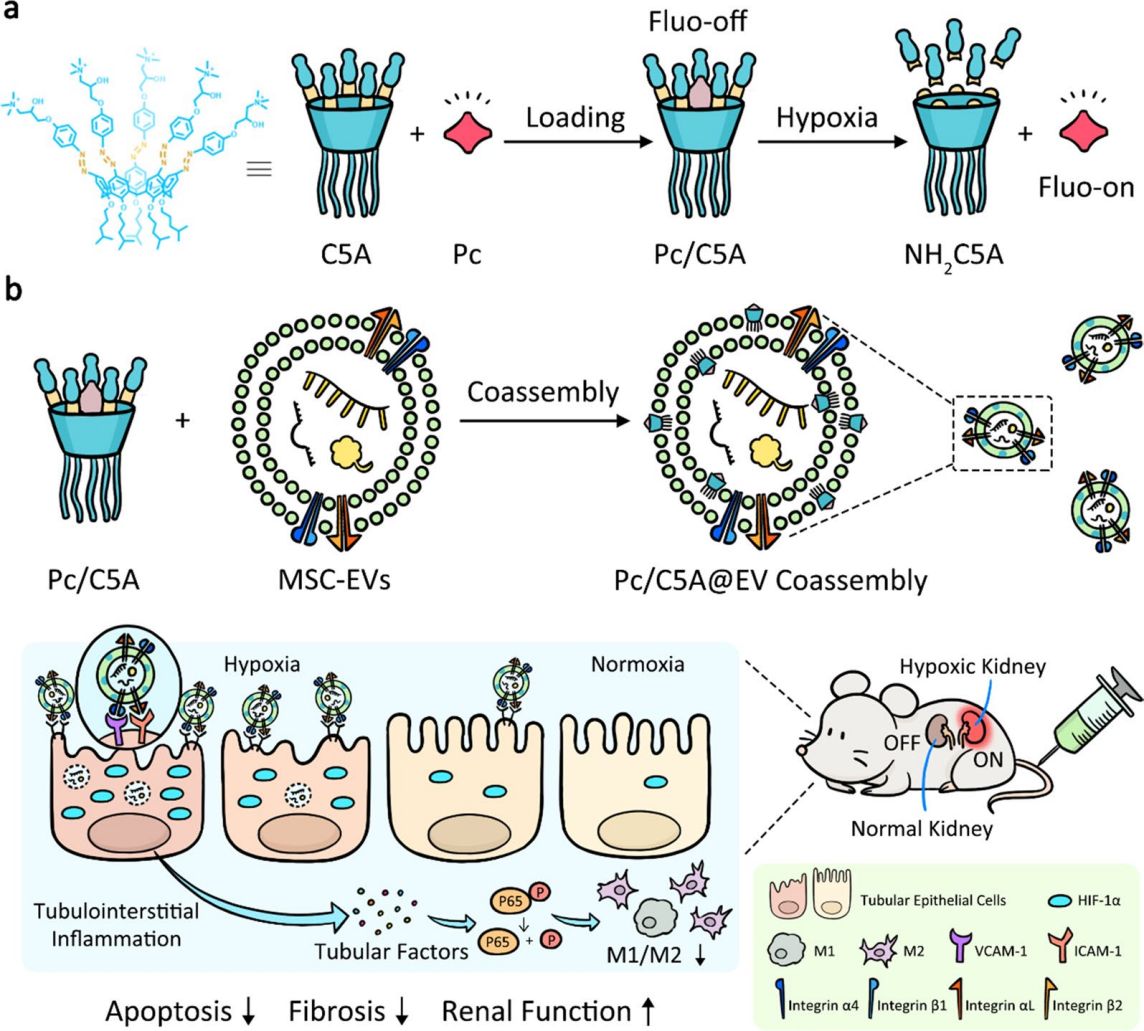
Schematic illustration of Pc/C5A@EV preparation (**a**) and simultaneous hypoxia-sensitive imaging and therapy in the injured kidneys (**b**)

Herein, we report the first example of a ternary hypoxia-sensitive coassembly composed of macrocyclic amphiphile, MSC-EVs and a commercially available dye. This nano-sized coassembly simultaneously accomplished renal-specific hypoxia imaging and targeted therapy in murine kidney hypoxia models. The commercially available near-infrared dye sulfonated aluminum phthalocyanine (Pc) was encapsulated in the cavity of quaternary ammonium-modified azocalix[5]arene pentaisohexyl ether (C5A) to form the hypoxia-sensitive host–guest complex Pc/C5A. Benefiting from the positive charge and amphiphilic features of C5A, the Pc/C5A complex was coassembled into MSC-EVs to construct the desired Pc/C5A@EV coassembly. In vivo, the coassembly specifically accumulated in the hypoxic kidneys, where it released Pc and achieved noninvasive hypoxia-sensitive imaging. Furthermore, the coassembly inhibited the apoptosis and fibrosis of the kidney, and promoted renal repair by inhibiting HIF-1α expression in TECs and inducing the M1-to-M2 macrophage transition through the NF-κB signaling pathway (Scheme 1).

## Materials and methods

### Materials

All chemicals and reagents were purchased from Sigma-Aldrich unless other-wise specified, and used without further purification. Alanine was purchased from Tokyo Chemical Industry. Glycine, glucose, creatinine and sodium dithionite (SDT) were purchased from J&K Chemical. Sodium dihydrogen phosphate was purchased from 3A chemistry. Valine was purchased from J&K Across. Lysine was obtained from Meryer. Sodium chloride was purchased from Benchmark. Bovine serum albumin (BSA) was purchased from Mreda. Glutathione was purchased from Energy Chemical. Sodium Nitrite (NaNO_2_), adenosine diphosphate (ADP), adenosine monophosphate (AMP), nicotinamide adenine dinucleotide (NAD) and glutamine were purchased from Aladdin. Glycidyl trimethylammonium chloride was purchased from Adamas Reagent. Pc (sulfonated aluminum phthalocyanine) was obtained from Frontier Scientific. Dulbecco's modified eagle's medium (DMEM)/F12, Roswell park memorial institute (RPMI) 1640 medium, fetal bovine serum (FBS), antibiotics (penicillin–streptomycin) and phosphate buffered saline (PBS) were purchased from Gibco. Distilled deionized water was obtained from a Millipore Milli-DI water purification system. Primary antibodies: anti-GAPDH (1:10,000), anti-HIF-1α (1:1000), anti-phosphorylated NF-κB p65 (1:1000), anti-NF-κB p65 (1:1000), GM130 (1:1000), CD63 (1:1000), ALIX (1:1500), CD47 (1:1000), ICAM-1 (1:1000), VCAM-1 (1:1000), integrin α4 (1:1000), αL (1:1000), β1 (1:1000), β2 (1:1000), iNOS (1:200), CD206 (1:50), F4/80 (1:200) were all from Abcam; CD9 (1:1000) was from Cell Signaling Technology. Secondary antibody (1:5000) were from System Bioscience. The West Pico Chemiluminescent Substrate Kits used to visualize protein signals were from Pierce. RIPA protein lysis buffer, Trizol and SYBR green regent were all from Invitrogen. LysoTracker Red and Alexa Fluor 594 phalloidin were purchased from Thermo Fisher.

### Synthesis of C5A

**Fig. 1:**
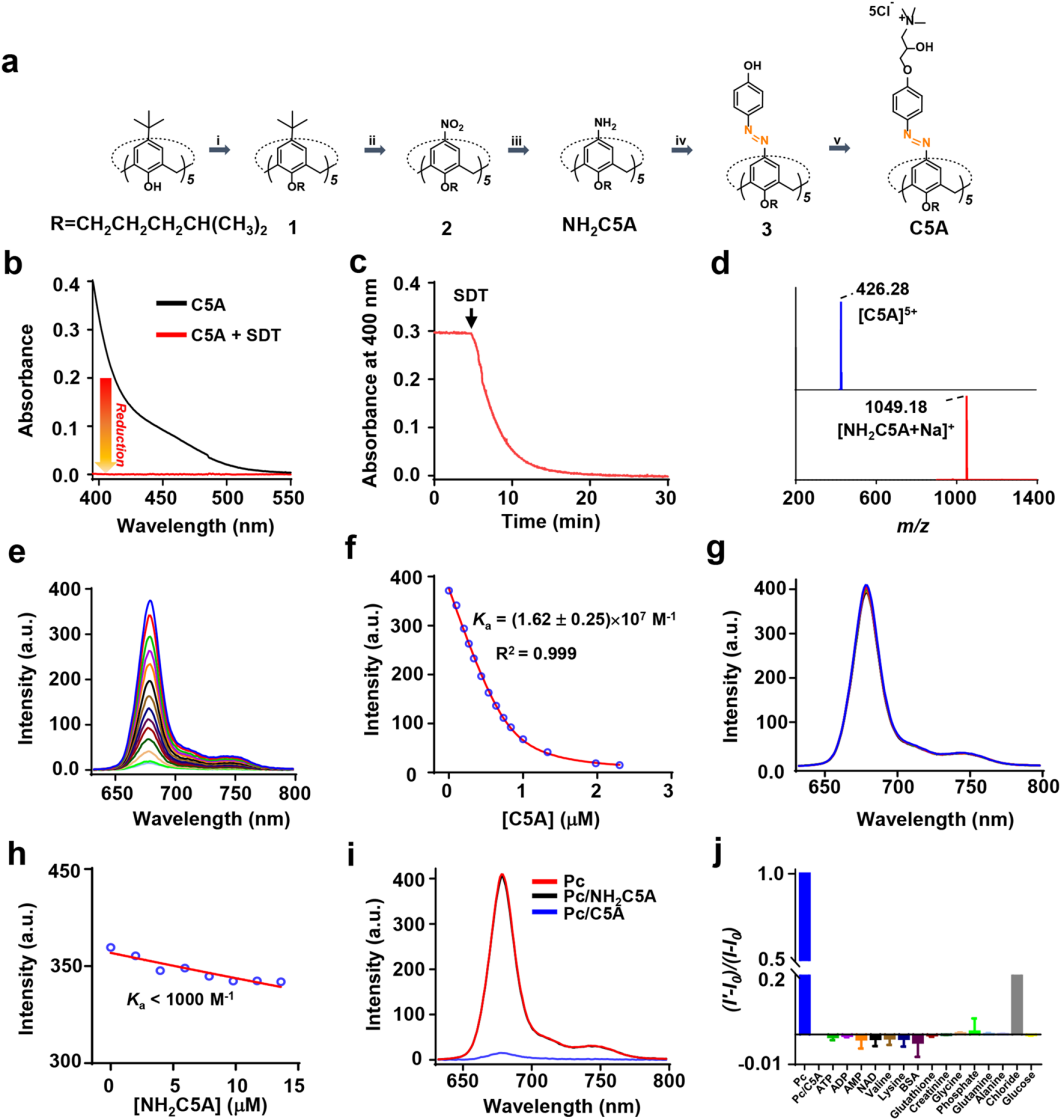
Synthesis, hypoxia response and molecular recognition of C5A. **a** C5A synthesis route. (i) K_2_CO_3_, RBr, CH_3_CN, reflux, yield 72%; (ii) AcOH, HNO_3_, dry CH_2_Cl_2_, 25 °C, yield 46%; (iii) SnCl_2_•2H_2_O, C_2_H_5_OH/AcOEt (1:1, v/v), reflux, yield 52%; (iv) NaNO_2_, HCl (aq), 0 °C; phenol, pyridine in tetrahydrofuran (THF), 25 °C, yield 64%; (v) isopropanol, glycidyltrimethylammonium chloride, reflux, yield 82%. **b** Absorbance of C5A (10 µM) before or after being reduced by SDT (2 mM). **c** C5A (10 µM) absorbance at 400 nm as a function of time following the addition of SDT (2 mM). **d** Mass spectra of C5A (10 µM) before (top) and after (bottom) incubation with SDT (2 mM). **e** Direct fluorescence titration of Pc (0.8 µM, *λ*_ex_ = 606 nm, *λ*_em_ = 678 nm) with C5A (up to 2.3 µM) and **f** the associated titration curve fitting according to a 1:1 binding stoichiometry. **g** Direct fluorescence titration of Pc (0.8 µM, *λ*_ex_ = 606 nm, *λ*_em_ = 678 nm) with NH_2_C5A (up to 13.62 µM) and **h** the associated titration curve fitting according to a 1:1 binding stoichiometry. The changes in fluorescence emission were too small to obtain a reasonable association constant. Therefore, the constant was estimated to be less than 1,000 M^−1^ according to the fitting equation [50]. **i** Fluorescence spectra of Pc (0.8 µM, *λ*_ex_ = 606 nm, *λ*_em_ = 678 nm), Pc/NH_2_C5A (0.8 and 2 µM, respectively), and Pc/C5A (0.8 and 2 µM, respectively). **j** Fluorescence responses of Pc/C5A (2 µM each) upon addition of biologically coexisting species in blood. *I* is the fluorescence of Pc alone, and *I*_0_ is the fluorescence of Pc/C5A, and *I*’ is the fluorescence of Pc/C5A upon addition of the species. The experiments in **b**-**j** were performed at 25 °C in PBS (pH = 7.4, 10 mM)

C5A was synthesized as shown in Fig. 1a. Briefly, the lower edge of the parent *p-tert*-butylcalix[5]arene was alkylated to attain compound **1**, then all *tert*-butyl groups were replaced with nitro groups to generate compound **2**. The nitro groups were then reduced to amino groups to obtain NH_2_C5A, and compound** 3** was obtained by diazotization. Finally, the target C5A receptor was obtained through a ring-opening reaction.

### Hypoxia response of C5A

We used ultraviolet-visible (UV–Vis) spectrometry, mass spectrometry and fluorescence spectrometry to investigate the hypoxia response of C5A. SDT was injected dropwise into 2.5 mL of C5A solution in a quartz cuvette under hypoxic conditions to monitor the reduction kinetics of C5A by UV–Vis spectrometry. The reduction product was analyzed with a mass spectrometer. In order to ensure the loaded probe can be readily unloaded by reducing C5A to NH_2_C5A, C5A and NH_2_C5A was injected dropwise into 2.5 mL of Pc solution in a quartz cuvette, respectively, and the fluorescence changes caused by host–guest complexation were monitored.

### Molecular recognition of C5A with Pc

When direct fluorescence titrations were performed, a mixed solution containing known concentrations of the C5A and Pc was injected dropwise into 2.5 mL of Pc solution in a quartz cuvette. Pc concentrations in the mixed solution and cuvette were kept constant during titration. Fluorescence intensity was measured before the first addition and after every addition until it reached a plateau. The fitting of data from direct host–guest titrations were performed in a nonlinear manner, and the fitting modules were downloaded from the website of Prof. Nau’s group (http://www.jacobs-university.de/ses/wnau) under the column of “Fitting Functions”. *K*_a_ was obtained by fitting the fluorescence intensity and total host concentration data according to a 1:1 host–guest binding stoichiometry. All fluorescence experiments were performed in PBS (10 mM, pH 7.4) at 25 °C.

### Fluorescence responses of Pc/C5A upon addition of various biologically coexisting species

Various biological coexisting species of blood was added separately to Pc/C5A (2/2 µM) in PBS, and stirred for 30 min to monitor the fluorescence intensity of Pc. The fluorescence of Pc alone was used as a control. The biological coexisting species and their concentrations used in these experiments were as follows: ATP 0.4 µM, ADP 0.1 µM, AMP 10 nM, NAD 24 µM, glutamine 0.5 mM, alanine 0.4 mM, valine 0.2 mM, glycine 0.3 mM and lysine 0.2 mM; phosphate 0.8 mM, chloride 95 mM, BSA 10 µg/mL, glutathione 8 µM, creatinine 80 µM and glucose 5 mM. The concentrations of all of the above components refer to their concentrations in human blood [38–40].

### Cell culture and MSC-EV isolation

Human umbilical cord-derived MSCs between the third and eighth passages were used in the experiments. MSCs and TEC cell line HK-2 was cultured in DMEM/F12 medium. The mouse macrophage cell line RAW264.7 was cultured in RPMI 1640 medium [35].

MSC-EVs were isolated as we previously described [33, 41]. EVs were collected from the culture supernatant that obtained from the medium containing 10% EV-depleted FBS for 48 h, centrifuged at 500 × g for 10 min to remove the cells, and then centrifuged at 2000 × g for 20 min and 5000 × g for 30 min to remove apoptotic bodies and cell debris. The resulting supernatant was then filtered using 0.22 µm filters and harvested by centrifugation in a SW32 Ti rotor for 2 h at 130,000 × g. The harvested precipitate was resuspended in PBS and then ultracentrifugated at 130,000 × g for another 2 h to remove contaminating proteins.

### The coassembly of Pc/C5A@EVs

Pc/C5A (10 and 20 µM, respectively) were added to 100 µg of MSC-EVs suspended in PBS and incubated for 2 h of at 37 °C. Then, the mixture was ultracentrifuged at 130,000 × g in PBS for 2 h. The supernatant containing the unassembled Pc, C5A, or Pc/C5A was removed, and the obtained pellet (Pc/C5A@EVs) was dissolved in PBS again. To block the integrins, Pc/C5A@EVs were incubated with 50 µg/mL of anti-integrin β1 and β2 or IgG at 4 °C overnight.

### Analysis of the fluorescence intensity of Pc/C5A@EVs in serum

Pc (10 µM), Pc/C5A (10 and 20 µM, respectively), and Pc/C5A@EVs (10 and 20 µM, respectively; MSC-EVs, 100 µg) were dissolved in 500 µL of serum in microtubes before fluorescence imaging (λ_ex_ = 606 nm, λ_em_ = 660–700 nm) with an IVIS Lumina imaging system. In the Pc + EV and Pc/C5A + EV groups, Pc or Pc/C5A was mixed with the MSC-EVs, and the ultracentrifugation was not performed.

### The stability of Pc/C5A@EVs

The stability of Pc/C5A@EVs was first examined using a UV–Vis spectrophotometer at different time points. 100 µg of MSC-EVs dissolved in 400 µL of PBS was added and incubated with Pc/C5A (10 and 20 µM, respectively) for 2 h, followed by ultracentrifugation at 130,000 × g for an additional 2 h. The supernatant containing free Pc/C5A and the Pc/C5A@EVs pellets were then gathered. The absorption of supernatants and Pc/C5A@EVs diluted in 3 mL of PBS were measured via UV spectrometry. Then, the stability of coassembly was characterized in PBS or 10% serum in PBS for 72 h by DLS and zeta potential assay. The morphology of Pc/C5A@EVs was further observed by transmission electron microscope (TEM) up to 72 h to verify the Pc/C5A@EV stability.

### The hypoxic response of Pc/C5A@EVs in vitro

A humidified atmosphere containing 5% CO_2_ was used as a normoxic cell culture condition. The hypoxic cell culture environment was adjusted by using a purging gas mixture (94% N_2_, 5% CO_2_, 1% O_2_). HK-2 cells (1 × 10^5^ cells per well) were seeded and cultured in confocal imaging chambers. After adherence, the cells were incubated with Pc/C5A@EVs (Pc/C5A at 10 and 20 µM, respectively; MSC-EVs: 100 μg) or Pc/C5A (10 and 20 µM, respectively) at 37 °C for 12 h under normoxic or hypoxic conditions, respectively. PKH26@EVs served as a reference. Subsequently, the cells were fixed in 4% paraformaldehyde (PFA; Sangon Biotech, Shanghai, China) for 10 min, washed with PBS three times, and then imaged using CLSM. Cell nuclei were counterstained with DAPI for 10 min. The cytoskeleton was stained with phalloidin.

### Animal models

All animal studies were performed in accordance with the Regulations for the Administration of Affairs Concerning Experimental Animals (Tianjin, revised in June 2004) and adhered to the Guiding Principles in the Care and Use of Animals of the American Physiological Society, and were approved by the Animal Ethics Committee of Nankai University (Tianjin, China). Experiments were conducted using 6-to 8-week-old male C57BL/6 mice purchased from Vital River Laboratory Animal Technology (Beijing, China). The mice were housed in a temperature-controlled sterile animal facility on a 12 h light/dark cycle, and the mice had ad libitum access to food and water. The hypoxic renal injury mouse models were established as described previously [5]. Mice were anesthetized and were then placed on a 37 °C heating plate. A unilateral hypoxic injury was induced by clamping the right renal pedicle for 45 min, and bilateral kidneys hypoxia injury was induced in mice by renal pedicle clamping for 35 min, then the clamps were released. The sham mice were administered with exposure of kidneys but without clamping the renal pedicle.

### Pc/C5A@EV tracking in mouse models

The hypoxic renal injury mice were randomly injected with 100 *µ*L of Pc/C5A (10 and 20 µM, respectively) or Pc/C5A@EVs (10 and 20 µM, respectively; MSC-EVs: 200 µg) (*n* = 5) into the tail vein. The IVIS Lumina imaging system was used to perform the fluorescence imaging, and exposing them to the following: Pc: λ_ex_ = 606 nm and λ_em_ = 660–700 nm or PKH26: λ_ex_ = 565–605 nm and λ_em_ = 565 nm at 10 nm. Scans were performed 1, 6, 12, 24, 48, and 72 h after injection. Exposure time = 200 ms. Ex vivo fluorescence images were acquired at different time points after sacrificing the mice and isolating their main organs.

### Detection of azo reductase in different organs

The tissues of liver, heart, spleen, lungs, normal kidney and hypoxic kidney were harvested and rinsed with PBS for protein extraction. After the addition of cell lysis buffer to each group, the mixture was centrifuged at 5000 × g for 5 min and then the supernatant was collected. 10 µmol of NADPH and 8 nmol of orange II were added to each group respectively. The concentration of azo reductase was spectrophotometrically assayed at 513 nm at room temperature [42].

### Histology and immunohistochemistry

The mice were killed on days 1, 3, and 7 postadministration, and the kidneys were excised. Some kidney tissue was fixed overnight in 4% PFA, embedded in paraffin, sectioned at 5 µm, and stained with H&E. The slices were examined using an optical microscope (Olympus BX51, Japan). The remaining kidney tissue fraction was embedded in OCT compound (Sakura Finetek) and cut into frozen sections (5 µm) for immunofluorescence staining. The renal tubules were stained with fluorescently tagged LTL. Tubulointerstitial inflammation was evaluated by immunostaining with antibodies against F4/80^+^, iNOS^+^ (M1 marker) and CD206^+^ (M2 marker).

### Flow cytometry analysis

TECs were incubated with Pc/C5A@EVs (10 and 20 µM, respectively; MSC-EVs, 100 µg) or Pc/C5A (10 and 20 µM, respectively) for 36 h under hypoxia to inhibit the secreted tubular factors, then the conditioned media of TECs was collected and cultured with the macrophages for 24 h. The obtained single-cell suspension was stained with anti-CD86-FITC (1:100, Elabscience) and anti-CD206-APC (1:100, Elabscience). Untreated macrophages were used as a negative control. The flow cytometry was performed on a FACSCalibur flow cytometer (BD Biosciences, NY, USA), and the data were analyzed using FlowJo software (TreeStar, Ashland, OR, USA).

### Statistical analysis

An independent *t*-test was used for two-group comparisons, and one-way ANOVA was used for multiple-group comparisons with a suitable post hoc test. Differences were considered significant as *P* < 0.05. All statistical analyses were employed to analyze the differences using GraphPad Prism 7.0 software (GraphPad software, Inc., San Diego, CA). All data are expressed as the mean ± S.D. (standard deviation).

## Results and discussion

### Synthesis and characterization of the hypoxia-sensitive Pc/C5A complex

We designed the hypoxia‐sensitive azocalixarene C5A based on the negative charge characteristic of MSC-EVs. C5A was synthesized as shown in Fig. 1a. Briefly, synthesis initiated from the parent *p-tert*-butylcalix[5]arene that is alkylated in its lower edge to attain compound **1**, bearing the conical conformation [43, 44]. Afterward, compound **1** was treated by AcOH and HNO_3_, replacing all *tert*-butyl groups with nitro groups via the isopropyl nitration reaction, to generate compound **2**. The nitro groups were then reduced to amino groups through the addition of SnCl_2_•2H_2_O in ethyl acetate and ethanol to obtain NH_2_C5A. Afterward, HCl and NaNO_2_ solutions were added for diazotization, and phenol was added to obtain compound **3**. Finally, the target C5A receptor was obtained through a ring-opening reaction between glycidyltrimethylammonium chloride and compound** 3** (Additional file 1: Fig. S1–3). The C5A design is well suitable for our purpose due to the following reasons: First, it is ready to be embedded into MSC-EV membranes as a cationic amphiphile through hydrophobic and electronic interactions. Among the calixarenes, we employed C5A because its cavity size ensured good binding properties [45, 46]. Second, the introduction of azobenzene generated a deep cavity that imparts a strong binding affinity towards various therapeutic and imaging agents [22, 23]. Moreover, azobenzene is hypoxia-sensitive [21]. In a hypoxic microenvironment, C5A is reduced to NH_2_C5A, releasing the cavity-loaded cargo in a controllable manner [23].

We used UV–Vis spectrometry and mass spectrometry to investigate the reduction of C5A after adding sodium dithionite (SDT), the proxy of azoreductase [47]. Azo absorption disappeared in 20 min after SDT was added, indicating that all five azo groups of C5A were completely reduced (Fig. 1b). The reduction kinetics were quantified by monitoring the absorbance at 400 nm in real time (Fig. 1c). The intensity attenuation curve was well fitted to a quasi-first-order reaction decay model (R^2^ = 0.998), giving a rate constant of 0.342 ± 0.012 min^−1^ (Additional file 1: Fig. S4). The half-life was 122 ± 4 s, which is at the same level as similar compounds containing a single azo group [48]. We further detected the reduction product of C5A via mass spectrometry. The mass spectrum of C5A showed a peak at 426.28, corresponding to [M]^5+^ (Additional file 1: Fig. S5). NH_2_C5A was detected following the incubation with SDT, as indicated by a peak at 1049.18, corresponding to [M + Na]^+^ (Fig. 1d).

Pc was used as the imaging probe because of its near-infrared light absorption and emission, high quantum yield, good photostability, low photobleaching and, more importantly, its strong binding with C5A [49]. The quaternary ammonium groups at the upper rim of C5A donate multiple salt bridge interactions (charge-assisted hydrogen bonds) with the sulfonate groups of Pc. The upper rim of C5A was modified with azophenyl groups which possess π − stacking interactions with the aromatic scaffold of Pc. Moreover, the introduction of azobenzene generated a deep hydrophobic cavity that imparts high binding affinities towards various therapeutic and imaging agents as proved in our previous works [23, 24]. The synergistic effect among several interactions results in the strong binding between C5A and Pc. The 1:1 binding affinity between C5A and Pc was determined to be (1.62 ± 0.25) × 10^7^ M^−1^ (Fig. 1e, f). Complexation-induced quenching by calixarenes is involved in the photoinduced electron transfer mechanism [50, 51]. Super-quenching is highly desired for hypoxia-sensitive imaging because it generates a relatively weak background. The reduced host NH_2_C5A was expected to bind weakly with Pc to achieve hypoxia-triggered release. We found that the binding affinities showed a tremendous Pc binding selectivity difference between C5A and NH_2_C5A, exceeding four orders of magnitude, thus confirming the predicted hypoxia response (Fig. 1g, h). Moreover, as shown in Fig. 1i, Pc fluorescence intensity was remarkably quenched by C5A (96%), while the addition of NH_2_C5A hardly changed Pc fluorescence because no appreciable host–guest complexation occurred. Thus, the loaded probe can be readily unloaded by reducing C5A to NH_2_C5A, resulting in hypoxia-selective imaging with a high signal-to-background ratio. Unlike covalent imaging methods [20], the Pc/C5A reporter pair may face competitive complexation by interfering substances in biological environments which can lead to Pc release and give rise to imaging noise. Therefore, we tested Pc/C5A fluorescence intensity changes in the presence of biologically coexisting species in blood. Pc fluorescence showed no apparent change after addition of the biological species described in Fig. 1j, demonstrating a satisfactory anti-interference ability of the Pc/C5A reporter pair for in vivo bioimaging as a result of their strong binding.

### Construction and characterization of the Pc/C5A@EV coassembly

**Fig. 2:**
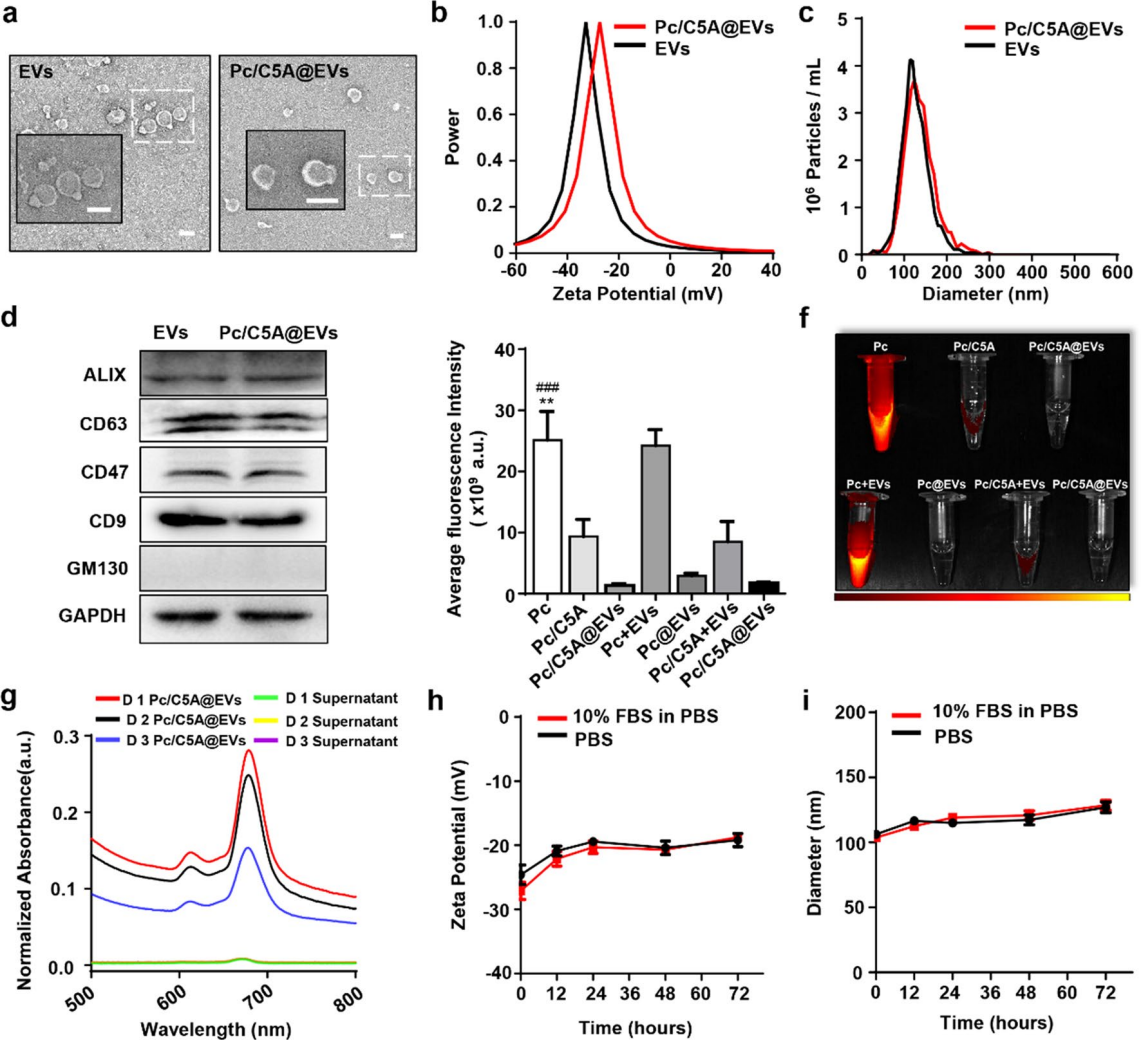
Characterization of the Pc/C5A@EV coassembly. **a** Representative TEM micrographs for EVs and Pc/C5A@EVs. Scale bar, 200 nm. **b** Zeta potential detection of EVs (black) and Pc/C5A@EVs (red). **c** Measurement of the size distribution of EVs (black) and Pc/C5A@EVs (red) by NTA. **d** Western blot analysis of GM130 and EV-specific biomarkers in EVs and Pc/C5A@EVs. **e** Quantitative fluorescence intensity analysis of the images in **f** (*n* = 5; ^**^ *P* < 0.01 compared with Pc/C5A; ^###^ *P* < 0.05 compared with Pc/C5A@EVs). **f** Fluorescence images of free Pc (10 µM), Pc/C5A (10 and 20 µM, respectively), Pc/C5A@EVs (10 and 20 µM of Pc and C5A, respectively; MSC-EVs, 100 µg), Pc + EVs (10 µM of Pc mixed with 100 µg of MSC-EVs without ultracentrifugation), and Pc/C5A + EVs (10 and 20 µM of Pc and C5A, respectively, and mixed with 100 µg of MSC-EVs without ultracentrifugation) in serum. **g** The stability of Pc/C5A@EVs in PBS at various time points measured by UV–Vis spectrometry. **h, i** The zeta potential and mean size changes of Pc/C5A@EVs incubated in PBS and PBS with 10% FBS for 72 h (*n* = 5)

After characterizing the Pc/C5A complex, we constructed the Pc/C5A@EV coassembly. As we previously reported, by serial ultracentrifugation, MSC-EVs were obtained from the culture media of MSCs [33, 41]. Subsequently, MSC-EVs were incubated with Pc/C5A at 37 °C for 2 h, and then subjected to ultracentrifugation in PBS for another 2 h to remove the unbound components. Both EVs and Pc/C5A@EVs appeared as round- to oval-shaped double-membrane vesicles in TEM micrographs (Fig. 2a). Successful loading of positively charged Pc/C5A into EVs was evidenced by the higher zeta potential of Pc/C5A@EVs (Fig. 2b). Moreover, the addition of SDT decreased the zeta potential, demonstrating the occurrence of a reductive reaction in the Pc/C5A@EVs (Additional file 1: Fig. S6). The size distribution and concentration of Pc/C5A@EVs and EVs were measured by nanoparticle tracking analysis (NTA), and the mean sizes of Pc/C5A@EVs and EVs were almost identical at approximately 120 nm, which was in agreement with the TEM results (Fig. 2c). The EV markers were then examined via Western blot. The presence of the cytosolic protein ALIX and surface protein CD63, and the absence of GM130 (a Golgi-derived contaminant) demonstrated the purity of our EV preparation [52]. Importantly, the expression of CD47 [53, 54] and CD9 [55] was confirmed in Pc/C5A@EVs, which also demonstrated the effective coassembly (Fig. 2d). We further examined the fluorescence intensity of Pc, Pc/C5A, and Pc/C5A@EVs in serum (Fig. 2e, f). In contrast to free Pc, Pc/C5A and Pc/C5A@EVs exhibited almost no detectable emission due to the robust complexation of Pc with C5A (Fig. 2f, upper part). Moreover, our results showed that the ultracentrifugation procedure removed all unbound Pc or Pc/C5A from EVs (Fig. 2f, lower part).

The stability of the Pc/C5A@EV coassembly was then examined over time via UV–Vis spectrometry. The results shown in Fig. 2g indicated that Pc/C5A was stably assembled into EVs for 72 h. Moreover, the zeta potential and size of Pc/C5A@EVs remained almost unchanged after incubation in PBS or PBS with 10% FBS for 72 h (Fig. 2h, i). TEM images showed that Pc/C5A@EVs remained the cup-shaped morphology up to 72 h (Additional file 1: Fig. S7). The loading efficiency of Pc/C5A@EVs was then measured by preparing the standard Pc/C5A solutions with known concentrations. A standard curve was established by detecting the fluorescence signal of Pc/C5A with a microplate reader, and the average loading efficiency of Pc/C5A was calculated as 11.7%. Furthermore, colocalization of Pc/C5A@EV fluorescence with that of LysoTracker Red was not observed (Additional file 1: Fig. S8), suggesting the evasion of lysosomal trapping via the CD9 protein in MSC-EVs. These findings suggest the long-term blood circulation potential of Pc/C5A@EV coassembly in vivo.

### In vitro imaging of TECs under hypoxia via Pc/C5A@EVs

**Fig. 3:**
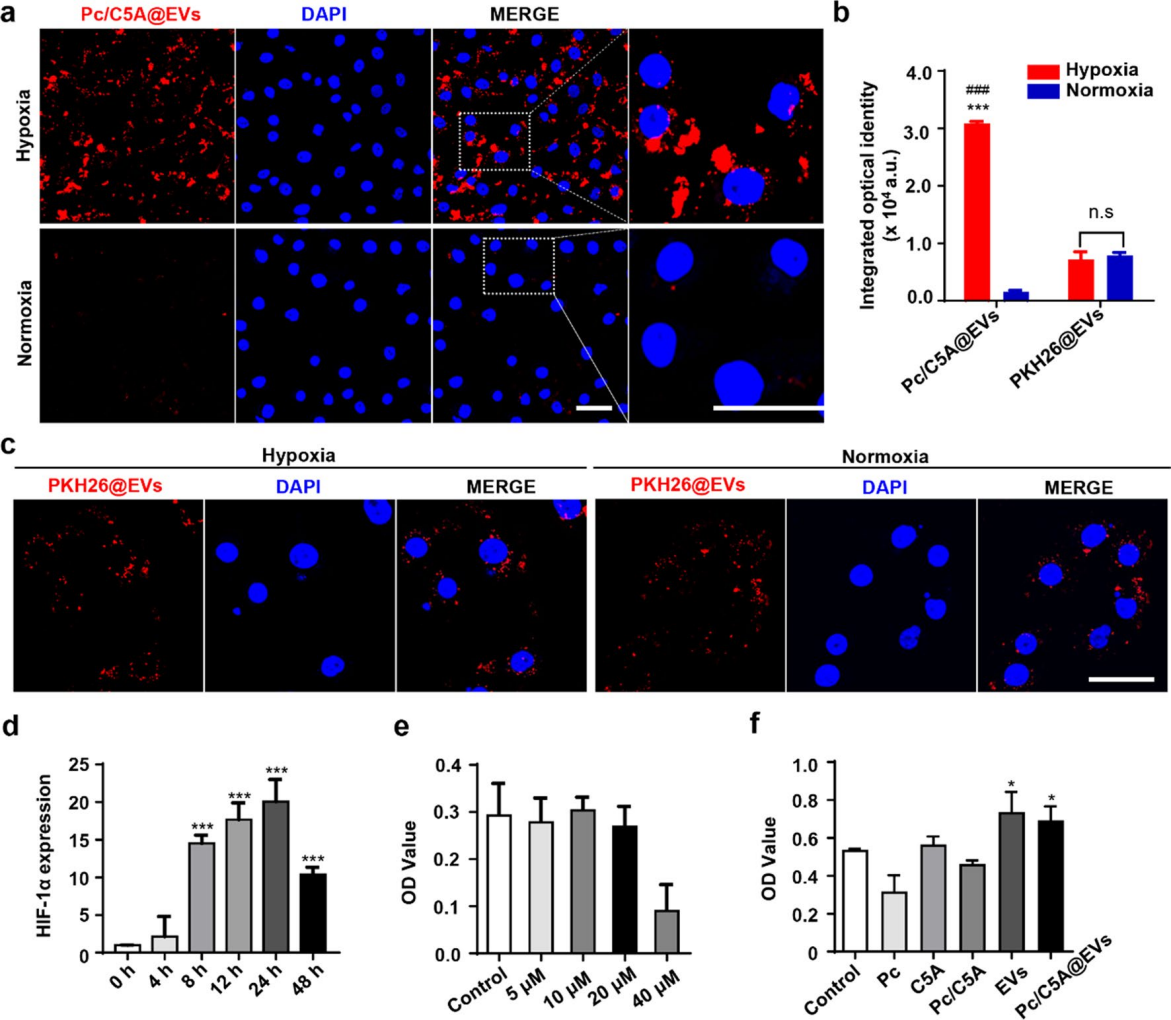
Hypoxia-sensitive imaging of TECs via Pc/C5A@EVs in vitro. **a** Representative CLSM images of TECs incubated with Pc/C5A@EVs under hypoxia or normoxia. Pc/C5A@EVs: red; DAPI: blue. Scale bars = 50 µm. **b** Quantitation of the CLSM micrographs (*n* = 5; ^***^ *P* < 0.01 compared with Pc/C5A@EVs under normoxia; ^###^ *P* < 0.01 compared with PKH26@EVs under hypoxia; n.s, not significant). **c** Representative CLSM images of TECs incubated with PKH26@EVs under hypoxia or normoxia. PKH26@EVs: red; DAPI: blue. Scale bars = 50 µm. **d** RT-qPCR analysis of the *HIF-1α* mRNA expression in TECs incubated in a hypoxic environment (*n* = 5; ^***^ *P* < 0.01 compared with 0 h. **e** MTT cell viability assay assessing cell toxicity of C5A at concentrations ranging from 5 µM to 40 µM. **f** MTT cell viability assay assessing the cell toxicity following treatment with Pc, 10 µM; C5A, 20 µM; Pc/C5A, 10 and 20 µM, respectively; MSC-EVs, 100 µg; and Pc/C5A@EVs, Pc/C5A, 10 and 20 µM, respectively; MSC-EVs, 100 µg. *n* = 5; ^*^ *P* < 0.05 compared with control

The hypoxia-sensitive imaging capacity of Pc/C5A@EVs was next evaluated in vitro. TECs are the leading cell type in the kidney cortex with the greatest vulnerability to hypoxic injury [3]; therefore, we evaluated the response of Pc/C5A@EVs to TECs under hypoxia. TECs were cultured under hypoxia or normoxia, and then 100 µg of Pc/C5A@EVs was added for an additional incubation of 12 h. As shown by confocal laser scanning microscopy (CLSM) in Fig. 3a, TECs incubated with Pc/C5A@EVs under normoxia exhibited almost undetectable red fluorescence signals upon excitation at 606 nm due to the superior quenching of Pc in C5A. In contrast, considerable red Pc/C5A@EV fluorescence signals were concentrated in the cytoplasm and exhibited high colocalization under hypoxia, indicating that Pc/C5A@EVs were effectively taken up by TECs, and the fluorescence was recovered by the hypoxia-sensitive release of Pc. The fluorescence signal in TECs under hypoxia was approximately 11.41-fold higher than that under normoxia after Pc/C5A@EV treatment (Fig. 3b). Additionally, Pc/C5A exhibited a cellular uptake and hypoxic response similar to that of Pc/C5A@EVs on account of the suitable quenching of Pc in C5A (Additional file 1: Fig. S9). However, free Pc without C5A showed no fluorescence signals under either condition (Additional file 1: Fig. S10). The commercial cell membrane labeling probe, PKH26 was subsequently coassembled into MSC-EVs as a reference to further verify the response of Pc/C5A@EVs to hypoxia in TECs. As shown in Fig. 3b and c, PKH26@EVs exhibited comparable fluorescence signals in TECs under normoxia and hypoxia, demonstrating that PKH26 cannot fulfill the hypoxia-sensitive imaging as Pc/C5A does.

The mRNA levels of *HIF-1α* were also detected via real-time quantitative polymerase chain reaction (RT-qPCR) in TECs under hypoxia to corroborate the hypoxic microenvironment. As shown in Fig. 3d, *HIF-1α* mRNA expression was upregulated at 8 h, and that level was sustained for up to 24 h, confirming a typical cellular hypoxic response. Finally, as shown in Fig. 3e C5A under 20 µM showed almost no cell toxicity in MTT assay; moreover, Pc/C5A and Pc/C5A@EVs exhibited good cell biocompatibility in vitro (Fig. 3f). The above in vitro data demonstrated that our hypoxia-sensitive coassembly can be sufficiently internalized by TECs, and the near-infrared fluorescence of Pc is adequately quenched by C5A under normoxia; however, the fluorescence emission is capable of being selectively activated under hypoxia*.*

### Safety evaluation of Pc/C5A@EVs

Encouraged by the ideal hypoxia-sensitive and biocompatible characteristics of Pc/C5A@EVs that were observed in vitro, we proceeded to explore their behaviors in vivo*.* First, safety evaluation was performed in healthy male C57BL/6 mice. The plasma hemolysis assay results confirmed the in vivo biocompatibility of Pc/C5A@EVs. As shown in Additional file 1: Fig. S11, Pc/C5A@EVs displayed a very low hemolysis ratio, which was below the allowable limit (5%) [56].

**Fig. 4:**
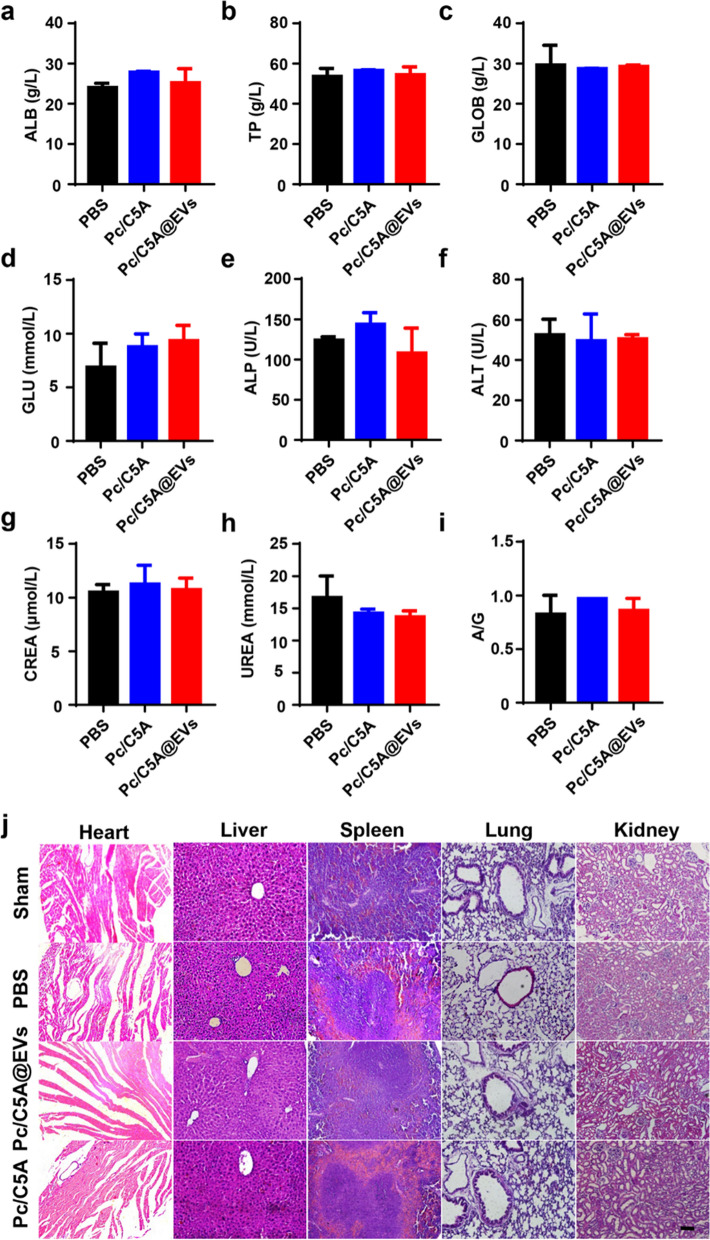
Safety evaluation of Pc/C5A@EVs. **a**–**i** Blood chemistry analysis of Pc/C5A and Pc/C5A@EVs in healthy mice (*n* = 5). ALB (**a**), TP (**b**), GLOB (**c**), GLU (**d**), ALP (**e**), ALT (**f**), CREA (**g**), UREA (**h**), A/G (**i**). **j** Typical H&E-staining images of heart, liver, spleen, lung, and kidney slices after different treatments in healthy mice (*n* = 3). Scale bar, 100 µm

Afterward, blood samples were collected on day 7 after intravenous injection of 0.1 mL of PBS, Pc/C5A (10 and 20 µM, respectively) or Pc/C5A@EVs (10 and 20 µM, respectively; MSC-EVs, 100 µg). No significant changes were detected in the albumin (ALB), total protein (TP), globulin (GLOB), glucose (GLU), alkaline phosphatase (ALP), alanine aminotransferase (ALT), creatinine [57] and urea (UREA) concentrations or in the albumin-globulin ratio (A/G) in the Pc/C5A-treated and Pc/C5A@EV-treated mice compared with the PBS-treated mice (Fig. 4a–i). Additionally, hematoxylin–eosin (HE)-stained slices showed that Pc/C5A@EV and Pc/C5A injections rarely resulted in any lesions in vital organs of normal mice (Fig. 4j). Moreover, no body weight loss was detected in different groups Additional file 1: Fig. S12). These results demonstrated that the Pc/C5A@EV coassembly is safe for in vivo applications and does not negatively affect any major organs.

### In vivo imaging of kidney hypoxia via Pc/C5A@EVs

Next, noninvasive fluorescence imaging was performed at designated time intervals after 0.1 mL of Pc/C5A@EVs (10 and 20 µM, respectively; MSC-EVs, 100 µg) or Pc/C5A (10 and 20 µM, respectively) was intravenously injected to the murine models of hypoxic renal injury induced by unilateral or bilateral ischemia/reperfusion (Additional file 1: Fig. S13a**)** [5, 58].

**Fig. 5:**
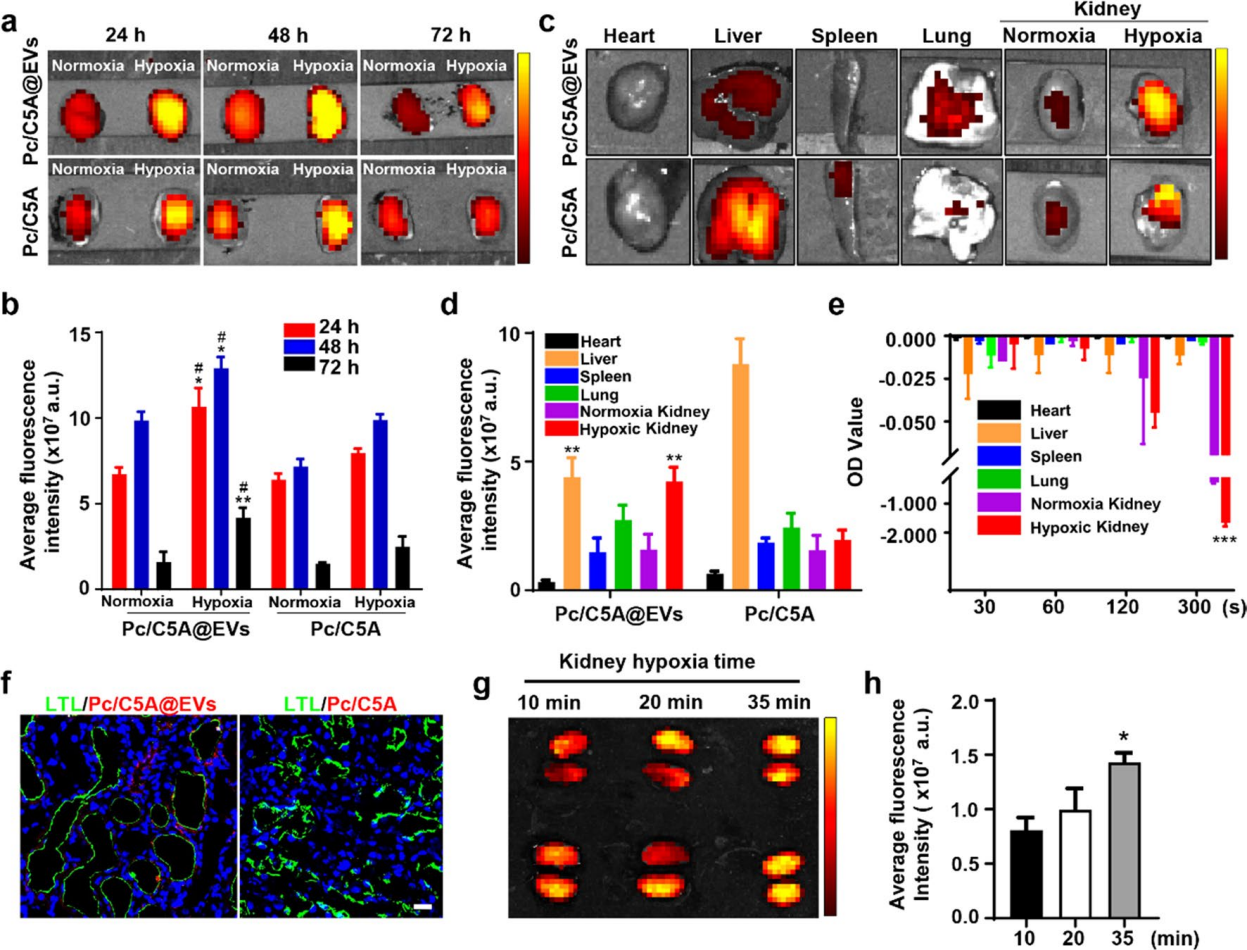
In vivo imaging of kidney hypoxia via Pc/C5A@EVs. **a** Ex vivo images of normoxic and hypoxic kidneys at designated time points after intravenous injection of Pc/C5A@EVs or Pc/C5A in mice with unilateral hypoxic renal injury. **b** Time-dependent fluorescence intensity changes in normoxic and hypoxic kidneys after Pc/C5A@EV or Pc/C5A injection (*n* = 5; ^*^P < 0.05 compared with Pc/C5A@EV-treated normoxic kidneys; ^**^P < 0.01 compared with Pc/C5A@EV-treated normoxic kidneys; ^*#*^*P* < 0.05 compared with Pc/C5A-treated hypoxic kidneys). **c** Ex vivo images of major organs in the Pc/C5A@EV or Pc/C5A group. **d** Fluorescence intensities in the major organs after sacrifice of the hypoxic renal injury mice on day 3 after injection (*n* = 5, ^**^P < 0.01 compared with Pc/C5A). **e** The expression of azo reductase in different organs (*n* = 5, ^***^P < 0.01 compared with normoxia kidney). **f** CLSM micrographs of kidney slices from the mice injected by Pc/C5A@EVs or Pc/C5A for 24 h. LTL: green; Pc/C5A, Pc/C5A@EVs: red; DAPI: blue. Scale bars = 20 µm. **g, h** Fluorescence imaging of the mice at 12 h postinjection after 10-, 20-, or 35-min bilateral hypoxic renal injury

In mouse models of unilateral hypoxic renal injury, fluorescence signals were quickly observed in hypoxic kidneys 1 h after injection of Pc/C5A@EVs or Pc/C5A; however, the contralateral normal kidneys showed almost no fluorescence signals (Additional file 1: Fig. S13b). The fluorescence intensity in the Pc/C5A@EV group was stronger than that in the Pc/C5A group, reached a maximum at 24 h postinjection and gradually subsided but was still visible at 72 h postinjection. However, fluorescence signals were undetectable in the Pc/C5A group by 72 h. Ex vivo imaging of dissected kidneys at 24, 48, and 72 h postinjection confirmed remarkable fluorescence accumulation in hypoxic kidneys in the Pc/C5A@EV group due to the specific hypoxia turn-on and targeting effects (Fig. 5a). The signals from the hypoxic kidneys in the Pc/C5A@EV group were 1.59-, 1.31-, and 2.64-fold stronger than those in the normal kidneys (Fig. 5b). Ex vivo fluorescence intensity in vital organs at 72 h postinjection revealed that the liver was the dominant organ in which the fluorescence signals accumulated in the Pc/C5A group. In contrast, in the Pc/C5A@EV group, the hypoxic kidney exhibited fluorescence signals comparable to those in the liver (Fig. 5c, d), which was consistent with the high expression of azo reductase in the supernatants of hypoxic kidneys among different organs (Fig. 5e) [42]. As references, imaging results after Pc, Pc@EV, or PKH26@EV administration showed rare fluorescence was visible in hypoxic kidneys up to 72 h postinjection (Additional file 1: Fig. S14). Ex vivo analysis of the major organs illustrated that fluorescence signals were present in both kidneys at 1, 24, and 72 h, and the highest fluorescence occurred in the livers (Additional file 1: Fig. S15). Subsequently, CLSM imaging of kidney tissue sections was conducted to localize the hypoxia-sensitive fluorescence signals. Cytoskeletal staining confirmed colocalization of Pc/C5A@EVs with hypoxic renal cells (Additional file 1: Fig. S16). In particular, the fluorescence signals of Pc/C5A@EVs were concentrated around the proximal tubules stained green with lotus tetragonolobus lectin (LTL), which was attributed to the sensitivity of TECs to hypoxia injury; moreover, the accumulation of Pc/C5A@EVs was far superior to that of Pc/C5A due to the targeting and immune escape effects of MSC-EVs (Fig. 5f).

We further verified the imaging capacity of Pc/C5A@EVs to hypoxic regions in the kidney in mice with bilateral hypoxic renal injury. The fluorescence intensity of hypoxic kidneys in the Pc/C5A@EV group gradually increased with the duration of hypoxia time, demonstrating enhanced accumulation of Pc/C5A@EVs with aggravation of hypoxic microenvironments in the kidneys (Fig. 5g, h).

### The coassembly targeted to the hypoxic kidneys via integrin receptor α_4_β_1_ and α_L_β_2_ on MSC-EVs

**Fig. 6:**
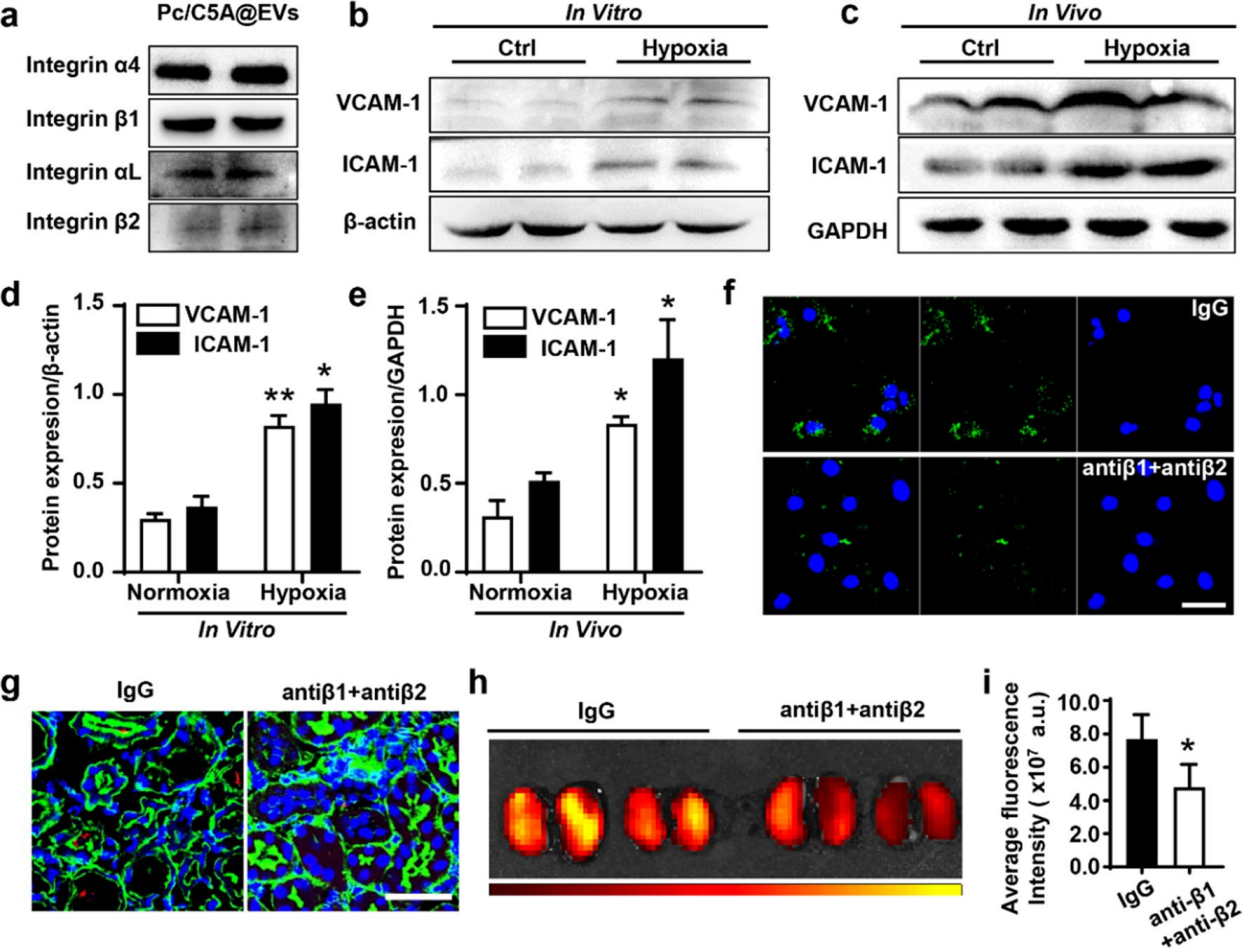
The coassembly targeted to the hypoxic kidneys via integrin receptor α4β1 and αLβ2. **a** Western blot of the integrin α4, αL, β1 and β2 expression in Pc/C5A@EVs. **b–e** Western blot of the VCAM-1 (**b**, **d**) and ICAM-1 (**c**, **e**) expression in TECs or renal tissues under hypoxia. (*n* = 5, ^*^P < 0.05 compared with normoxia group; ^**^P < 0.01 compared with normoxia group) **f**, **g** Representative CLSM images of the cellular uptake of Pc/C5A@EVs after blocking of integrin β1 and β2 in hypoxic TECs or renal tissues. Scale bars = 50 µm. **h**, **i** Fluorescence imaging and quantitative analysis of IgG- or blocking antibody-treated Pc/C5@EVs in bilateral hypoxic renal injury mice. (*n* = 5; ^*^P < 0.05 compared with the IgG group)

Previous studies have reported that the interactions between integrin-α4β1 and vascular cell adhesion molecule 1 (VCAM-1) or integrin-αLβ2 and intercellular cell adhesion molecule-1 (ICAM-1) are involved in specific adhesion of EVs to inflamed tissues [57]. As shown in Fig. 6a, Western blot results showed that integrin-α4β1 and integrin-αLβ2 were expressed in Pc/C5A@EVs. Moreover, the upregulated expression of VCAM-1 and ICAM-1was detected in hypoxic TECs and kidney tissues by Western blot (Fig. 6b–e). Results from Fig. 6f demonstrated that the incubation of anti-integrin-β1 and anti-integrin-β2 antibodies with Pc/C5A@EVs overnight inhibited the Pc/C5A@EV uptake in TECs under hypoxia. Moreover, the uptake and accumulation of Pc/C5A@EVs treated with anti-integrin-β1 and anti-integrin-β2 antibodies also decreased in the hypoxic kidney tissues, indicated by the reduced fluorescence signals detected by CLSM (Fig. 6g) and fluorescence imaging (Fig. 6h, i), demonstrating that integrin-α4β1 and integrin-αLβ2 present on Pc/C5A@EVs are responsible for their homing to hypoxic regions in the kidney.

Collectively, the above in vitro and in vivo results demonstrate that the as-designed Pc/C5A@EV coassembly has high specificity and displays remarkable ability for noninvasive and precise imaging of kidney hypoxia. Smart MSC-EVs specifically led Pc/C5A to the hypoxic kidney via adhesion molecules; moreover, they preserved Pc/C5A from rapid metabolism and improved its circulation time by exerting their native properties, thereby enabling Pc/C5A to achieve reinforced and desirable hypoxia turn-on imaging. With these properties, we offer a prospective approach for comprehensive tracing and evaluating kidney hypoxia.

### Pc/C5A@EVs promoted the renal recovery by inhibiting HIF-1α expression and tubulointerstitial inflammation

**Fig. 7:**
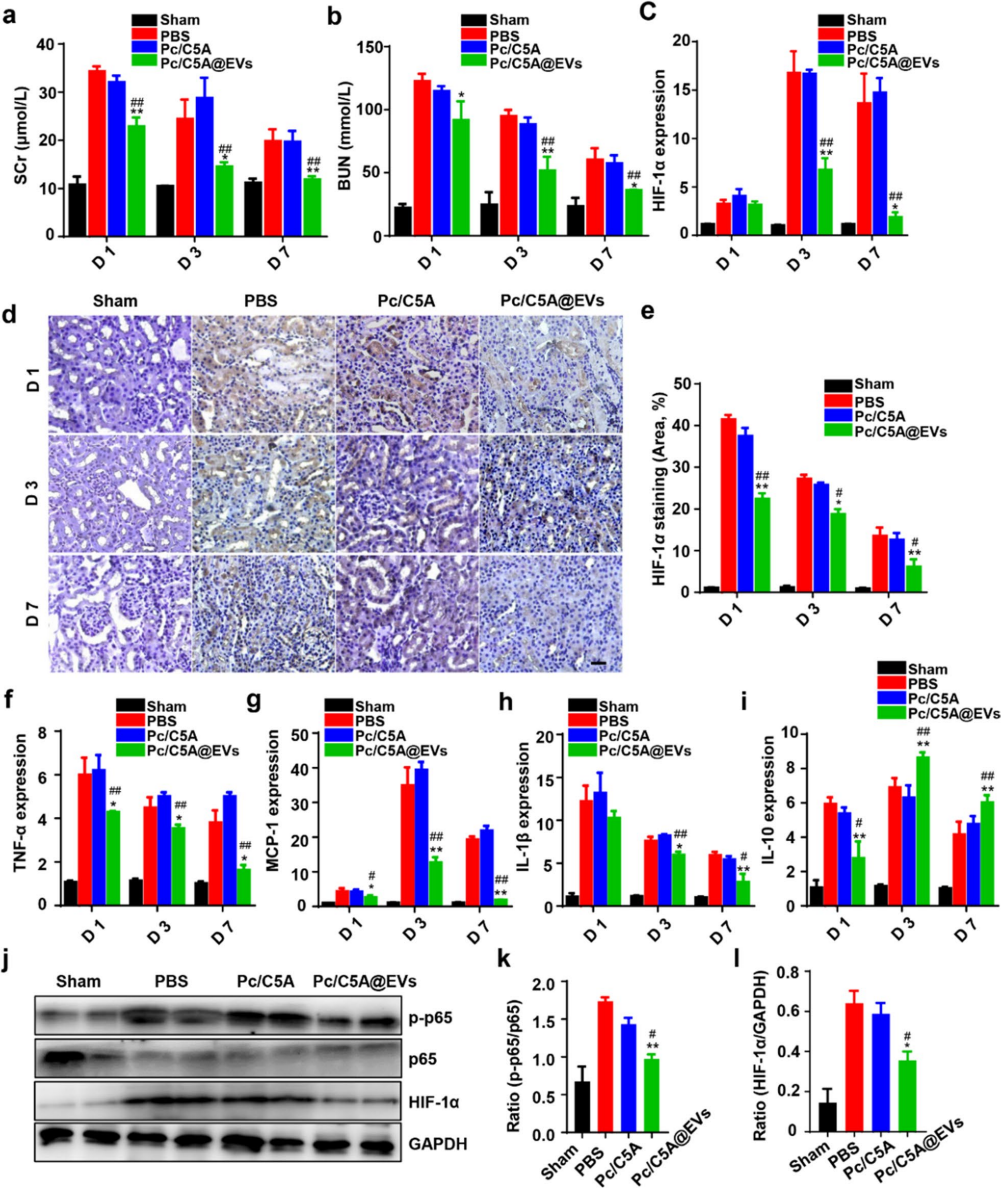
Pc/C5A@EVs downregulated the HIF-1α expression and NF-κB signaling pathway in mice with kidney hypoxia injury. **a**, **b** Measurement of SCr and BUN levels at different time points (*n* = 5; ^*^P < 0.05 compared with PBS; ^**^P < 0.01 compared with PBS; ^##^*P* < 0.01 compared with Pc/C5A). **c** RT-qPCR analysis of *HIF-1α* mRNA expression in different groups on days 1, 3, and 7 (*n* = 5; ^**^P < 0.01 compared with PBS; ^##^*P* < 0.01 compared with Pc/C5A; ^*^P < 0.05 compared with PBS. **d** Micrographs depicting HIF-1α immunostaining in renal tissues on days 1, 3, and 7 after injection. Scale bar = 100 µm. **e** HIF-1α staining quantitation in different groups (*n* = 5; ^**^P < 0.01 compared with PBS; ^##^*P* < 0.01 compared with Pc/C5A; ^*^P < 0.05 compared with PBS; ^#^*P* < 0.05 compared with Pc/C5A). **f**–**i** RT-qPCR detection of *TNF-α* (**f**), *MCP-1* (**g**), *IL-1β* (**h**) and *IL-10* (**i**) expression in renal tissues of different groups on day 7 (*n* = 5; ^*^P < 0.05 compared with PBS; ^**^P < 0.01 compared with PBS; ^#^*P* < 0.05 compared with Pc/C5A; ^##^*P* < 0.01 compared with Pc/C5A). **j–l** Western blot analysis of p-p65, p65 and HIF-1α expression in different groups (*n* = 5; ^**^P < 0.01 compared with PBS; ^#^*P* < 0.05 compared with Pc/C5A)

After validating the preferable imaging capacity of Pc/C5A@EVs in hypoxic kidneys, we explored the regenerative effects of Pc/C5A@EVs in mouse models of bilateral hypoxic renal injury. The results depicted in Fig. 7a and b revealed that serum creatinine (SCr) and blood urea nitrogen (BUN) were markedly upregulated in the PBS and Pc/C5A groups on days 1, 3 and 7 after injection, indicating decreased renal function in kidney hypoxia mice. However, renal function exhibited a significant improvement after Pc/C5A@EV administration. Histologically, protein casts and TEC injury were observed in renal injury mice, which was largely diminished by Pc/C5A@EV treatment (Additional file 1: Fig. S17). Moreover, Pc/C5A@ EV treatment decreased cell apoptosis (Additional file 1: Fig. S18). Compared with the levels in the PBS and Pc/C5A groups, the mRNA levels of apoptotic genes, including *CASP3, CASP9, CASP8, BAX,* and *BAD* were downregulated, which was consistent with reduced expression of the apoptotic protein Caspase 8 (Additional file 1: Fig. S19). Persistent hypoxia activated resident interstitial fibroblasts and led to interstitial fibrosis. Masson histological staining of renal sections performed at 4 weeks postinjection after bilateral ischemia/reperfusion injury showed that Pc/C5A@EV administration alleviated kidney fibrosis (Additional file 1: Fig. S20).

The HIF-1 family regulates central cellular responses to hypoxia [59]. During kidney hypoxia, HIF-1α is mainly expressed in TECs [60]. HIF-1α escapes destruction and translocates to the nucleus, where it activates downstream genes at different loci and induces endogenous cellular adaption against hypoxic stress [57]. However, constant activation of HIF-1α provokes inflammatory responses and exacerbates interstitial fibrosis [9, 10, 61]. Both RT-qPCR (Fig. 7c) and immunohistochemistry assessments (Fig. 7d, e) revealed that Pc/C5A@EVs significantly reduced HIF-1α expression on days 1, 3 and 7 after injection compared to the expression in the other groups. Meanwhile, tubulointerstitial inflammation was observed in kidney hypoxia mice on days 1, 3 and 7, as indicated by the increased mRNA expression of the pro-inflammatory cytokines tumor necrosis factor-α (*TNF-α*), monocyte chemoattractant protein-1 (*MCP-1*), and interleukin-1β (*IL-1β*) and the decreased mRNA levels of the anti-inflammatory cytokine interleukin-10 (*IL-10*), which were significantly overturned after Pc/C5A@EV transplantation (Fig. 7f–i). Our data suggested that the time-course inhibition of HIF-1α expression after Pc/C5A@ EV treatment was strongly linked with the downregulation of pro-inflammatory cytokines, indicating a cross-talk between HIF-1α expression and tubulointerstitial inflammation during kidney hypoxia as previously demonstrated [5, 60]. We continued to explore the signaling pathway linking hypoxia and inflammation. Previous publications demonstrated that HIF-1α mediates the pro-inflammatory nuclear factor κB (NF-κB) signaling pathway in a variety of pathological conditions, and persistent activation of endothelial HIF-1α participated in NF-κB-dependent hypertensive kidney injury [62, 63]. Accordingly, we detected the NF-κB signaling pathway in different groups on day 3 after injection. Western blot results revealed that Pc/C5A@EV administration inhibited the p65 phosphorylation to p-p65 (Fig. 7j, k), similar to the inhibition of HIF-1α protein (Fig. 7j, l).

### Pc/C5A@EVs induced M1-to-M2 macrophage transition by inhibiting HIF-1α expression in hypoxic TECs and the downstream NF-κB signaling pathway

In response to kidney hypoxia, macrophages are recruited to the interstitium by pro-inflammatory factors secreted by TECs and trigger tubulointerstitial inflammation. It is generally accepted that in contrast to pro-inflammatory M1 macrophages, M2 macrophages exert anti-inflammatory effects and promote renal regeneration [64, 65]. Consequently, we proceeded to investigate whether the inhibition of HIF-1α expression and tubulointerstitial inflammation induced by Pc/C5A@EVs was linked with macrophage transition. The fluorescence signal of Pc/C5A@EVs colocalized with that of macrophages stained green with F4/80 in kidney tissues of renal hypoxia-injured mice, whereas little fluorescence intensity was observed in the Pc/C5A group (Additional file 1: Fig. S21).

**Fig. 8:**
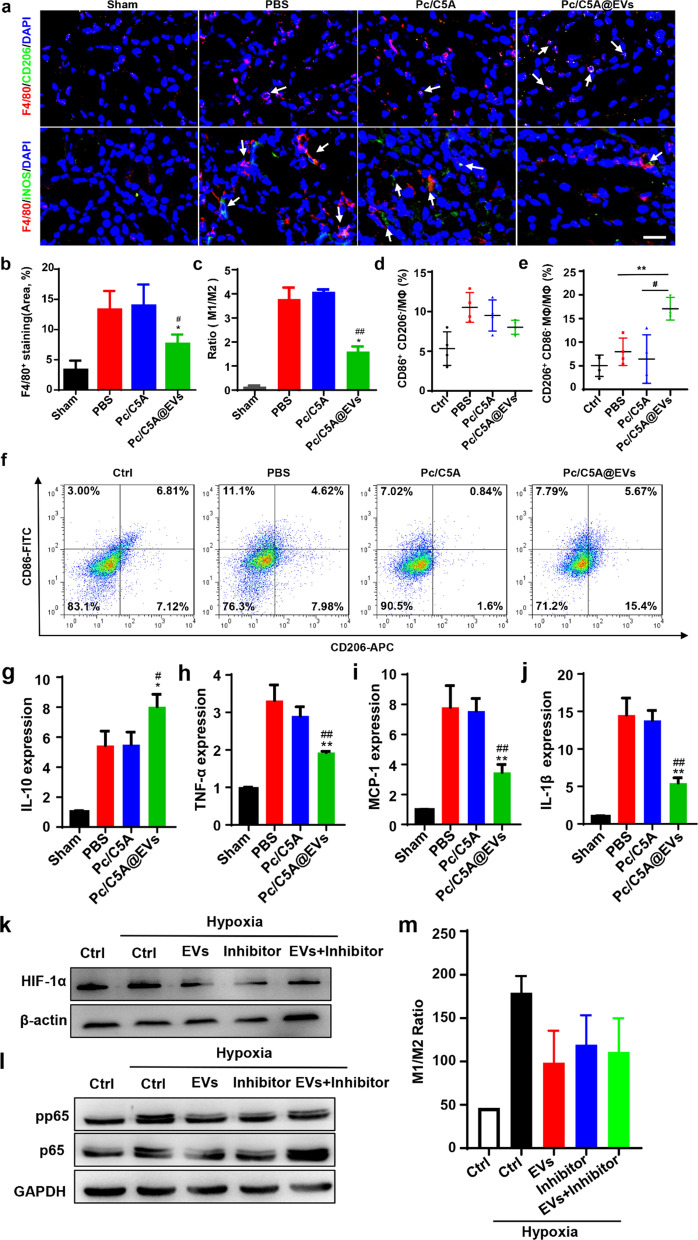
Pc/C5A@EVs induced M2 macrophage transition in hypoxic kidneys. **a** Immunofluorescence staining images of F4/80 (red), iNOS (green) and CD206 (green) in kidney tissues of different groups. DAPI: blue. Scale bar = 100 µm. **b** Quantitation of F4/80^+^ areas of kidney tissues. **c** Quantitative analysis of the M1-to-M2 ratio in kidney tissues. **d**–**f** Flow cytometry analysis showing the percentages of M1 (CD86^+^CD206^−^, (**d**) or M2 (CD206^+^CD86^−^, (**e**) Phenotypes in RAW 264.7 cells from different groups (*n* = 4, ^**^P < 0.01 compared with PBS; ^#^ *P* < 0.05 compared with Pc/C5A). **g**–**j** The mRNA levels of anti-inflammatory gene *IL-10* (**g**), pro-inflammatory genes *TNF-α* (**h**), *MCP-1* (**i**) and *IL-1β* (**j**) in RAW264.7 cells from different groups (*n* = 3; ^*^ *P* < 0.05 compared with PBS; ^**^P < 0.01 compared with PBS; ^#^*P* < 0.05 compared with Pc/C5A; ^##^*P* < 0.01 compared with Pc/C5A). **k** HIF-1α expression in different groups detected by Western blot. **l** p-p65 and p65 expression in different groups detected by Western blot. **m** Flow cytometry analysis showing the ratio of M1 (CD86^+^CD206^−^)/M2 (CD206^+^CD86^−^) in RAW 264.7 cells from different groups

Tubulointerstitial inflammation, which was reflected by upregulation of F4/80^+^ macrophage infiltration, was obviously present in the PBS and Pc/C5A groups on day 3 after injection and was significantly attenuated after Pc/C5A@EV administration (Fig. 8a, b). Moreover, F4/80^+^ macrophages in the Pc/C5A@EV group expressed decreased levels of inducible nitric oxide synthase (iNOS), an M1 marker, and elevated levels of CD206, an M2 marker, indicating an M1-to-M2 macrophage phenotypic transition (Fig. 8a, c).

To further confirm that Pc/C5A@EV treatment can induce the M1-to-M2 transition in kidney hypoxia, TECs were incubated with Pc/C5A@EVs or Pc/C5A for 36 h under hypoxia to inhibit the secreted tubular factors. Then, the conditioned medium of TECs was collected and cultured with macrophages for 24 h. Flow cytometry analysis revealed a significant higher percentage of CD206^+^ M2 macrophages, but a lower percentage of CD86^+^ M1 macrophages after treatment with Pc/C5A@EVs, and the opposite trend was observed in the PBS and Pc/C5A groups (Fig. 8d–f). Consistently, the mRNA levels of pro-inflammatory cytokines were decreased but the anti-inflammatory cytokine mRNA levels were increased by conditioned medium of TECs treated with Pc/C5A@EVs in vitro (Fig. 8g–j).

Finally, we explored whether the M2 macrophage transition mediated by Pc/C5A@EVs was linked to the inhibition of HIF-1α expression in TECs and defined the related signaling pathway. TECs were exposed to hypoxia for 36 h and treated with of HIF-1α inhibitor, Pc/C5A@EVs or their combination. Then, conditioned medium was collected and incubated with macrophages for 24 h. We found that culture with conditioned medium containing the Pc/C5A@EVs or the HIF-1α inhibitor efficiently inhibited HIF-1α expression in TECs and the activation of NF-κB signaling pathway in macrophages (Fig. 8k, l); moreover, flow cytometry analysis showed that the levels of CD86, the M1 macrophage marker, were significantly downregulated, whereas the levels of CD206, the M2 macrophage marker, were upregulated in macrophages, resulting a decreased M1/M2 ratio (Fig. 8m). Taken together, based on the in vivo and in vitro data, we concluded that Pc/C5A@EVs drove macrophage polarization towards the anti-inflammatory M2 phenotype in mice of hypoxic injury by inhibiting the HIF-α expression of TECs and the downstream NF-κB signaling pathway.

## Conclusions

In conclusion, we developed a novel, nanoscale hypoxia-sensitive Pc/C5A@EV coassembly, that can achieve both specific imaging of kidney hypoxia and targeted therapy for kidney injury. Complexation-induced fluorescence quenching endows the Pc/C5A complex with hypoxia-sensitive imaging activation, specifically triggered by azoreductase. Coassembly of Pc/C5A with MSC-EVs reduced the off-target effects and promoted the delivery efficiency. Consequently, the hybrid, ternary Pc/C5A@EV coassembly overcomes the challenge of noninvasive and precise imaging of kidney hypoxia in vivo, which cannot be achieved by any single component or any binary combination of the three. Moreover, Pc/C5A@EVs realize ideal renal repair by inhibiting HIF-1α expression in TECs and inducing the M1-to-M2 macrophage transition. We also identified the NF-κB signaling pathway to link hypoxia and inflammation in kidney injury.

We provide a supramolecular strategy for development of innovative theranostics for kidney hypoxia by making full use of molecular recognition and self-assembly. Of note, our strategy of coassembling supramolecular amphiphiles with MSC-EVs is potentially translational. To be envisaged, the strategy of coassembling endogenous cell-derived membranous structures and exogenous macrocyclic receptors is adaptable to diagnose and treat several diseases of concern [66], where the coassembling components can be easily tuned. One can conveniently incorporate these two species into a universal platform for personalized therapy as long as compatibility permits, specifically, any membrane with targeting, stealth, long circulation, and immune privilege [66–68], and any macrocyclic amphiphile with the ability to load and facilitate controlled release of treatment and imaging agents.

## Supplementary Information

**Additional file 1: Figure S1.** Synthetic route of C5A. **Figure S2.** ^1^H NMR spectrum of 1 in DMSO-d6, 400 MHz, 25 °C. **Figure S3.** (a) ^1^H NMR spectrum of **C5A** in DMSO-*d*_6_, 400 MHz, 25 °C. (b) ^13^C NMR spectrum of **C5A** in DMSO-*d*_6_, 100 MHz, 25 °C. **Figure S4.** Absorbance of C5A (10 µM) at 400 nm as a function of time following addition of SDT (2 mM) in PBS buffer (10 mM), and the corresponding fitting curve according to quasi-first order reaction decay model. **Figure S5.** Mass spectrum of C5A (QFT-ESI). **Figure S6.** Zeta potential detection of C5A@EVs and Pc/C5A@EVs upon incubation with or without SDT in PBS buffer. (a) Zeta potential measure of C5A@EVs in the absence (blue) or presence of SDT (red) in PBS buffer (10 mM, pH = 7.4) at 25 ℃. (b) Zeta potential measure of Pc/C5A@EVs in the absence (blue) or presence of SDT (red) in PBS buffer (10 mM, pH = 7.4) at 25 ℃. **Figure S7.** Representative TEM images of Pc/C5A@EVs on day 1, 3 and 7. Scale bar, 100 nm. **Figure S8.** In vitro colocalization of Pc/C5A@EVs and EVs with LysoTracker Red in TECs by CLSM. **Figure S9.** CLSM images of Pc/C5A under hypoxic or normoxic condition. **Figure S10.** CLSM images of Pc@EVs under hypoxic or normoxic condition. **Figure S11.** Hemolysis rate of Pc/C5A and Pc/C5A@EVs after incubation with mouse erythrocytes. **Figure S12.** Body weight changes of the mice with different treatments (n = 5). **Figure S13.** In vivo imaging of kidney hypoxia via Pc/C5A@EVs and Pc/C5A. (a) Illustration of the imaging tracing strategy after intravenous injection of Pc/C5A@EVs or Pc/C5A in unilateral hypoxic renal injury mice. (b) In vivo fluorescence images of Pc/C5A@EVs and Pc/C5A in unilateral hypoxic renal injury mice. **Figure S14.** In vivo imaging of hypoxia-injured mice with intravenous injection of Pc, Pc@EVs or PKH26@EVs at different time intervals (n = 3). **Figure S15.** (a) Ex vivo images of major organs at the designed time points after Pc, Pc@EV or PKH26@EV administration. (b-d) Time-dependent fluorescence intensity changes in major organs at designated time intervals after sacrificing renal hypoxia-injured mice (n = 5). **Figure S16.** Representative CLSM images of kidney slices from the renal hypoxia-injured mice after administration of Pc/C5A@EVs or Pc/C5A for 24 h. **Figure S17.** (a) Representative H&E-staining images of kidney sections on day 1, 3 and 7 after injection. Massive necrosis in the proximal tubules with hyaline cast formation (asterisks) was observed, and administration of Pc/C5A@EVs largely prevented histopathologic alterations after hypoxic injury. Scale bar, 50 µm. (b-c) Quantitative histological assessment of hyaline cast formation (b) and tubular necrosis (c) on day 3 postinjection**. Figure S18.** RT-qPCR analysis of the expression of apoptosis-related genes on day 7 postinjection. **Figure S19.** Representative Western blot showing Caspase 8 expression in kidney tissues with different treatments on day 7 postinjection. (a) Representative Western blot images of Caspase 8 in different groups. (b) Quantitative analysis of Western blot**. Figure S20.** Representative images of Masson trichrome staining in different groups (n = 8). **Figure S21.** Representative images of immunofluorescence staining of F4/80 (green) and Pc/C5A@EVs or Pc/C5A (red) in the kidney tissues in different groups. **Figure S22.** The raw data for Western blot in Fig. 2d. **Figure S23.** The raw data for Western blot in Fig. 6a. **Figure S24.** The raw data for Western blot in Fig. 6b. **Figure S25.** The raw data for Western blot in Fig. 6c. **Figure S26.** The raw data for Western blot in Fig. 7j. **Figure S27.** The raw data for Western blot in Fig. 8k-l. **Table S1** Primers used in the RT-qPCR assay.

## Data Availability

All data generated or analyzed during this study are included in this article.

## Abbreviations

EVs: Extracellular vesicles
MSC-EVs: Mesenchymal stem cell-excreted extracellular vesicles
Pc: Sulfonated aluminum phthalocyanine
AKI: Acute kidney injury
CKD: Chronic kidney disease
TECs: Tubular epithelial cells
HIF-1α: Hypoxia-inducible factor-1 alpha
TIF: Tubulointerstitial fibrosis
BOLD-MRI: Blood oxygen level-dependent magnetic resonance imaging
SDT: Sodium dithionite
PFA: Paraformaldehyde
VCAM-1: Vascular cell adhesion molecule-1
ICAM-1: Intercellular cell adhesion molecule-1
LPS: Lipopolysaccharide
DIO: 3,3’-Dioctadecyloxacarbocyanine perchlorate
H&E: Hematoxylin–eosin staining
TEM: Transmission electron microscopy
MTT: 3-(4, 5-Dimethylthiazol-2-yl)-2, 5-diphenyltetrazolium bromide
ALT: Alanine aminotransferase
AST: Aspartate aminotransferase
TP: Total protein
GLOB: Globulin
CHOL: Cholesterol
UA: Uric acid
CREA: Creatinine
UREA: Urea
GLU: Glucose
RT-qPCR: Quantitative real-time polymerase chain reaction
MSCs: Mesenchymal stem cells
SCr: Serum creatinine
BUN: Blood urea nitrogen
CLSM: Confocal laser scanning microscopy
NTA: Nanoparticle tracking analysis
