# Supplementary material for: Coassembly of hypoxia-sensitive macrocyclic amphiphiles and extracellular vesicles for targeted kidney injury imaging and therapy
Source: J Nanobiotechnology. 2021 Dec 27;19:451. doi: 10.1186/s12951-021-01192-w (PMC8714429; doi:10.1186/s12951-021-01192-w)
Supplement: Supplementary file 1 — Additional file 1: Figure S1. Synthetic route of C5A. Figure S2. 1H NMR spectrum of 1 in DMSO-d6, 400 MHz, 25 °C. Figure S3. (a) 1H NMR spectrum of C5A in DMSO-d6, 400 MHz, 25 °C. (b) 13C NMR spectrum of C5A in DMSO-d6, 100 MHz, 25 °C. Figure S4. Absorbance of C5A (10 µM) at 400 nm as a function of time following addition of SDT (2 mM) in PBS buffer (10 mM), and the corresponding fitting curve according to quasi-first order reaction decay model. Figure S5. Mass spectrum of C5A (QFT-ESI). Figure S6. Zeta potential detection of C5A@EVs and Pc/C5A@EVs upon incubation with or without SDT in PBS buffer. (a) Zeta potential measure of C5A@EVs in the absence (blue) or presence of SDT (red) in PBS buffer (10 mM, pH = 7.4) at 25 ℃. (b) Zeta potential measure of Pc/C5A@EVs in the absence (blue) or presence of SDT (red) in PBS buffer (10 mM, pH = 7.4) at 25 ℃. Figure S7. Representative TEM images of Pc/C5A@EVs on day 1, 3 and 7. Scale bar, 100 nm. Figure S8. In vitro colocalization of Pc/C5A@EVs and EVs with LysoTracker Red in TECs by CLSM. Figure S9. CLSM images of Pc/C5A under hypoxic or normoxic condition. Figure S10. CLSM images of Pc@EVs under hypoxic or normoxic condition. Figure S11. Hemolysis rate of Pc/C5A and Pc/C5A@EVs after incubation with mouse erythrocytes. Figure S12. Body weight changes of the mice with different treatments (n = 5). Figure S13. In vivo imaging of kidney hypoxia via Pc/C5A@EVs and Pc/C5A. (a) Illustration of the imaging tracing strategy after intravenous injection of Pc/C5A@EVs or Pc/C5A in unilateral hypoxic renal injury mice. (b) In vivo fluorescence images of Pc/C5A@EVs and Pc/C5A in unilateral hypoxic renal injury mice. Figure S14. In vivo imaging of hypoxia-injured mice with intravenous injection of Pc, Pc@EVs or PKH26@EVs at different time intervals (n = 3). Figure S15. (a) Ex vivo images of major organs at the designed time points after Pc, Pc@EV or PKH26@EV administration. (b-d) Time-dependent fluorescence intensity changes in major organ [file 12951_2021_1192_MOESM1_ESM.pdf]

Additional file 1

Coassembly of hypoxia-sensitive macrocyclic amphiphiles and extracellular vesicles for targeted kidney injury imaging and therapy

Yuan-Qiu Cheng[1†], Yu-Xin Yue[2†], Hong-Mei Cao[1,7†], Wen-Chao Geng[2], Lan-Xing Wang[1], Xin-Yue Hu[2], Hua-Bin Li[2], Qiang Bian[6], Xiang-Lei Kong[5], Jian-Feng Liu[7], De-Ling Kong[4], Dong-Sheng Guo[2,*], Yue-Bing Wang[1,3*]

[*]Correspondence: dshguo@nankai.edu.cn (D. Guo), wangyuebing@nankai.edu.cn (Y. Wang)

[†]Yuan-Qiu Cheng, Yu-Xin Yue and Hong-Mei Cao contributed equally to this work

[1] Nankai University School of Medicine, Tianjin 300071, China.

[2] College of Chemistry, Key Laboratory of Functional Polymer Materials (Ministry of Education), State Key Laboratory of Elemento-Organic Chemistry, Nankai University, Tianjin 300071, China.

[3] Tianjin First Central Hospital, School of Medicine, Nankai University

[4] The Key Laboratory of Bioactive Materials, Ministry of Education, College of Life Sciences, Nankai University, Tianjin 300071, China.

[5] College of Chemistry, State Key Laboratory of Elemento-Organic Chemistry, Nankai University, Tianjin 300071, China.

[6] National Pesticide Engineering Research Center, College of Chemistry, Nankai University, Tianjin 300071, China.

[7] Key Laboratory of Radiopharmacokinetics for Innovative Drugs, Chinese Academy of Medical Sciences, and Institute of Radiation Medicine, Chinese Academy of Medical Sciences & Peking Union Medical College, Tianjin 300192, China.

SUPPLEMENTARY SCHEMES AND FIGURES

1 Supplemental Methods

Apparatus.

NMR data were recorded on a Bruker AV400 spectrometer. Mass spectra were performed on an Autoflex III LRF200-CID (MALDI-TOF) and a Varian QFT-ESI (QFT-ESI) mass spectrometer. Ultraviolet-visible (UV-Vis) spectra were recorded in a quartz cell (light path 10 mm) on a Cary 100 UV-Vis spectrophotometer equipped with a Cary dual cell peltier accessory. The fluorescence measurements were recorded in a conventional quartz cell (light path 10 mm) on a Cary Eclipse equipped with a Cary single-cell peltier accessory. Nanoparticle tracking analysis (NTA) was carried out in ParticleMetrix-PMX. Dynamic light scattering (DLS) and zeta potential assays were performed on a laser light scattering spectrometer (BI-200SM) equipped with a digital correlator (TurboCorr). Transmission Electron Microscopy (TEM) were performed on a Talos F200C electron microscope (Thermo Fisher) at an acceleration voltage of 200 KV. Confocal laser scanning microscopy (CLSM) images were captured by Leica TSC SP8. EVs were harvested by ultracentrifugation in a SW32 Ti rotor (Beckman Coulter, L-100XP Ultracentrifuge, CA). Fluorescence Imaging was performed via an IVIS Lumina imaging system (Xenogen Corporation).

Mass Spectrometry Experiments to Test the Hypoxia-Responsiveness of C5A.

The mass spectra of C5A in PBS were measured by a Varian QFT-ESI (QFT-ESI)

mass spectrometer, and then SDT (2 mM) was added to C5A (10 μM) under hypoxic conditions and incubated for 20 min. The reduction product was analyzed with MALDI-TOF.

Fitting of the Kinetics of the Reduction.

The attenuation curve of the intensity was fitted in a quasi-first order reaction decay model ($A_t = A_0 e^{-kt}$). The fitting of the data gave a k value, and the value of half-life was calculated by ln2/k.

MSC-EV Characterization.

TEM was used for the visualization of MSC-EV and Pc/C5A@EV morphology. Samples were deposited on copper grids covered with a carbon support film and dried for 2 min at room temperature. The excess fluid was removed with a piece of filter, and samples were negatively stained with 2% uranyl acetate for 30 s. These samples were air-dried for 60 min to capture the images. The size distribution and concentration were measured by NTA.

Calculation of Pc/C5A Loading Content.

The OD value of Pc/C5A at different concentrations was measured via UV-vis spectroscopy at a given wavelength (680 nm) in 2.5 ml of PBS. The Beer-lambert law was used to quantify the concentration of Pc/C5A@EVs. Equations and standard curve

of Pc/C5A used to calculate concentration and loading content were as follows:

Standard curve of Pc/C5A based on UV-vis spectroscopy at 680 nm:

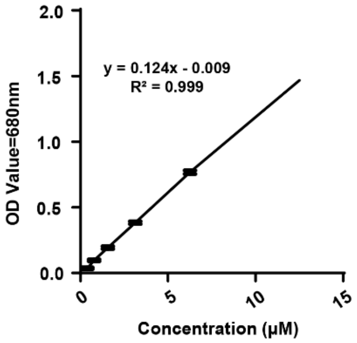

Concentration of Pc/C5A = (OD 680 / pathlength) × standard coefficient × dilution factor

Loading efficiency of Pc/C5A= [mPc/C5A / mPc/C5A@EVs] × 100%

***In Vitro* Colocalization of Pc/C5A@EVs and MSC-EVs with LysoTracker.**

Pc/C5A@EVs or MSC-EVs dissolved in 1 mL of PBS was incubated with 5 μM of DiO at 37 °C for 5 min. Then, the mixture was ultracentrifuged at 130,000 × g for another 2 h to remove unbound dyes and was resuspended in PBS again. Renal tubular epithelial cells (TECs, 5 × 10^3 cells per well) were seeded in climbing slices and cultured for 12 h. Then, TECs were renewed with the culture medium containing 100 μg of DiO-labelled Pc/C5A@EVs or MSC-EVs for another 6 h. 50 nM of fluorescent probe LysoTracker Red was added at 37 °C for 30 min. TECs were washed twice and then fixed in 4% paraformaldehyde for 10 min. Subsequently, they were counterstained with 4,6-diamidino-2-phenyiindole (DAPI) for 10 min and imaged via CLSM.

***In Vitro* Cytotoxicity Assay.**

TECs were seeded at a density of 5 × 10^3 cells per well in 96-well plates in DMEM/F12 medium. Pc (10 μM), C5A (5–20 μM), Pc/C5A (10 and 20 μM, respectively), MSC-EVs (100 μg), and Pc/C5A@EVs (Pc/C5A at 10 and 20 μM, respectively; 100 μg MSC-EVs) were added to cells. After 48 h of incubation, 20 μL of MTT solution (0.5 mg/mL) was added to each well, and the plates were cultured for an additional 4 h. The supernatant was then removed, and dimethyl sulfoxide (DMSO) was added to the wells to dissolve the formazan crystals. The absorbance of MTT at 490 nm was measured using a microplate reader (Biotek, VT, USA).

Safety Evaluation of Pc/C5A and Pc/C5A@EVs.

For hemolysis experiments, the fresh mouse blood was harvested and mixed with anticoagulant. The blood was centrifuged at 3000 rpm for 15 min to collected the red blood cells. The hemolytic reaction was conducted in 10 mL glass tubes at 37 °C after mixing equal volumes of Pc/C5A and Pc/C5A@EVs at different concentrations with erythrocytes. After 1 h of incubation, the mixed liquor was centrifuged 5 min at 5000 rpm. The optical density of the supernatant was measured at 570 nm. Absorbance of red blood cells lysed with 0.1% (by volume) Triton X-100 (assumed to be 100% hemolytic) was used as a positive control, and the negative control was normal saline. The hemolytic rates (RHR%) of Pc/C5A@EVs was calculated using the following formula[1, 2] :

$$\text{Hemolysis ratio (\%)} = (A_{sample} - A_{saline}) / (A_{water} - A_{saline}) \times 100\%.$$

To evaluate the safety of Pc/C5A and Pc/C5A@EVs, 12 male 5-6 week C57BL/6 were randomly divided into three groups (*n* = 3), followed by intravenous injection of 100 μL of PBS, Pc/C5A (10 and 20 μM, respectively), Pc/C5A@EVs (10 and 20 μM, respectively; MSC-EVs: 100 μg). All mice were sacrificed on day 7 after injection. Blood samples were collected for blood chemistry assay of the concentration of albumin (ALB), total protein (TP), globulin (GLOB), alkaline phosphatase (ALP), alanine aminotransferase (ALT), creatinine (CREA), urea (UREA), glucose (GLU) and albumin-globulin ratio (A/G). Major organs including the heart, liver, spleen, lungs and kidneys were collected and stained with hematoxylin-eosin (H&E) for histopathologic analysis. The mice were weighed every day to observe the dynamic mass change in each group.

Renal Function Analysis.

The bilateral hypoxic injury was induced in mice by clamping the bilateral renal pedicles for 45 min. Blood samples were collected from the orbital venous plexus at selected time points on days 1, 3, and 7 after injection and subjected to serum creatinine (SCr) and blood urea nitrogen (BUN) detection using a commercial kit (Jiancheng Biotech, Nanjing, China).

Real-Time Quantitative Polymerase Chain Reaction (RT-qPCR).

Total RNA was extracted from the tissue samples using TRIzol according to the

manufacturer's manual. mRNA was quantified by qRT-PCR with the QuantiTect SYBR Green PCR Kit and gene-specific primers. Supplementary **Table S1** lists all primers used for RT-qPCR.

Western Blot.

Western blot was performed as previously described [3]. The protein concentration in kidney tissues, cultured cells, and Pc/C5A@EVs was determined by BCA Protein Assay Reagent according to the manufacturer's instructions. Protein samples were lysed in ice-cold lysis buffer [150 mM NaCl, 50 mM Tris-HCl (pH 7.5), 1 mM EDTA, 1% NP-40, 0.1% SDS, 0.25% sodium deoxycholate, 1 mM phenylmethylsulfonyl fluoride (PMSF), 1 mM β-glycerophosphate, 1 mM NaF, 1mM Na_3VO_4, and protease inhibitor cocktail tablets (Roche Molecular Biochemicals, Indianapolis, IN, USA)]. Aliquots containing 30 μg of protein were mixed with a loading buffer and denatured at 95 °C for 5 min. Proteins were separated by 12% sodium dodecyl sulfate-polyacrylamide gel electrophoresis and transferred to polyvinylidene fluoride membranes. Membranes were blocked with 5% skim milk in Tris-buffered saline/Tween-20 (TBST) buffer [20 mM Tris-HCl (pH 7.5), 136 mM NaCl, and 0.1% Tween-20] and then probed overnight with primary antibodies. The membranes were then incubated with horseradish peroxidase-conjugated secondary antibodies. Protein signals were visualized using the West Pico chemiluminescent substrate kit.

2 Syntheses of C5A

5,11,17,23,29-Pentaamino-31,32,33,34,35-penta (4-methylpentloxy) calixarene (**NH2C5A**) were synthesized according to our previous literature [4]. The synthetic of C5A was illustrated as **Figure S1**.

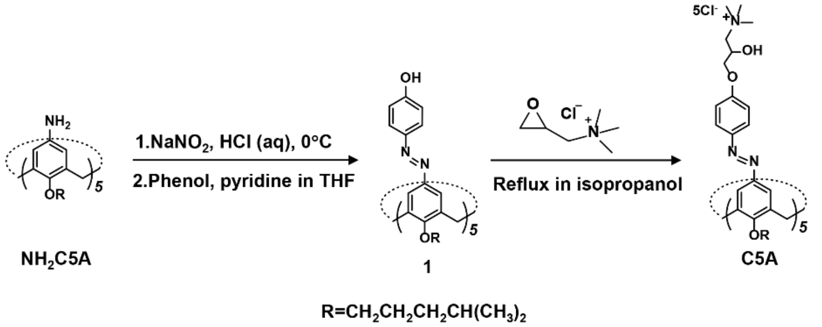

Figure S1. Synthetic route of C5A**.**

An aqueous HCl solution (1 M, 1.45 mL) was added to a solution of **NH2C5A** (171 mg, 0.17 mmol) in THF (10 mL) at 0 °C. A solution of $NaNO_2$ (73 mg, 1.06 mmol) in H_2O (5 mL) precooled to 0 °C was added slowly via a syringe. The reaction mixture was stirred at room temperature for 1 h. A solution of phenol (200 mg, 2.12 mmol) and triethylamine (0.2 mL, 1.45 mmol) in THF (4 mL) was added slowly at 0 °C. After 12 h at room temperature, the reaction mixture was poured slowly into H_2O (200 mL). The formed precipitate was collected by suction filtration and washed with water. After purification by column chromatography, a yellow solid of **1** was obtained (169 mg, 64%). ^{1}H NMR (400 MHz, DMSO-d_6, δ): 10.12 (s, 5H, OH), 7.60 (s, 10H, calix-H), 7.58 (d, J = 9.0 Hz, 10H, ArH), 6.78 (d, J = 9.0 Hz, 10H, ArH), 4.59 (d, J = 13.6 Hz, 5H, Ar-CH_2-Ar), 3.90 (t, J = 7.5 Hz, 10H, CH_2-O-Ar), 3.68 (d, J = 13.8 Hz, 5H, Ar-CH_2-Ar), 1.98 (m, 10H, -CH_2-CH_2-CH-), 1.65 (m, 5H, -CH-), 1.38 (m, 10H, -CH_2-CH_2-

CH-), 0.97 (d, J = 6.6 Hz, 30H, -$(CH_3)_2$) **(Figure S2).**

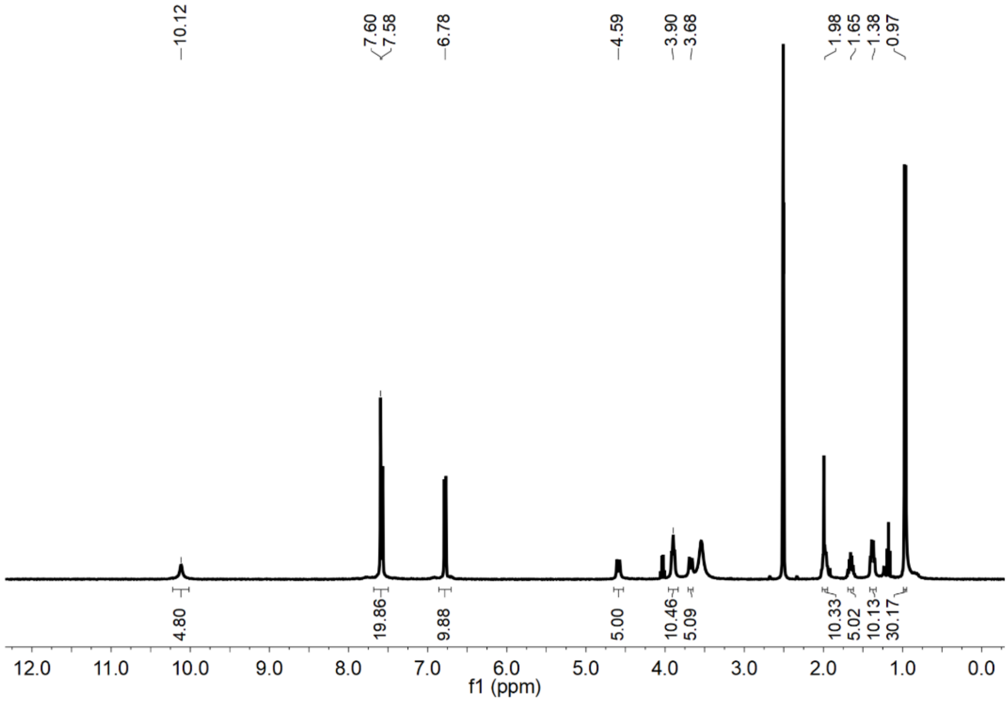

Figure S2. ^{1}H NMR spectrum of **1** in DMSO-d_6, 400 MHz, 25 °C.

Glycidyltrimethylammonium chloride (358 mg, 2.36 mmol) was added to a solution of **1** (390 mg, 0.25 mmol) in isopropanol (10 mL). The mixture was refluxed for 12 h and the solvent was removed in vacuo. The residue was dissolved in ethanol, then large amounts of acetone was added to obtain yellow solid of **C5A** (473 mg, 82%). ^{1}H NMR (400 MHz, DMSO-d_6, δ): 7.66 (d, J = 6.4 Hz, 10H, ArH), 7.59 (s, 10H, calix-H), 6.98 (d, J = 6.84 Hz, 10H, ArH), 6.23 (s, 5H, OH), 4.60 (d, J = 12.76 Hz, 5H, Ar-CH_2-Ar), 4.49 (s, 5H, CH), 4.01 (d, J = 18.72 Hz, 10H, OCH_2), 3.91 (m, 10H, OCH_2), 3.58 (m, 15H, NCH_2, Ar-CH_2-Ar), 3.21 (s, 45H, -$N(CH_3)_3$),1.95 (m, 10H, -CH_2-CH_2-CH-), 1.65 (m, 5H, -CH_2-CH_2-CH-), 1.38 (m, 10H, -CH_2-CH_2-CH-), 0.96 (d, J = 5.24 Hz, 30H, -$(CH_3)_2$).^{13}C NMR (100 MHz, DMSO-d_6, δ): 160.75, 157.77, 148.00, 146.86, 135.11, 124.68, 123.46, 115.44, 74.62, 70.91, 68.08, 64.21, 53.96, 35.10, 30.57, 29.68, 28.14,

22.99. MS (QFT-ESI): $[M]^{5+}$: m/z: calcd. for ($C_{125}H_{180}N_{15}O_{15}{}^{5+}$):426.2751, found 426.2751 (**Figure S3**).

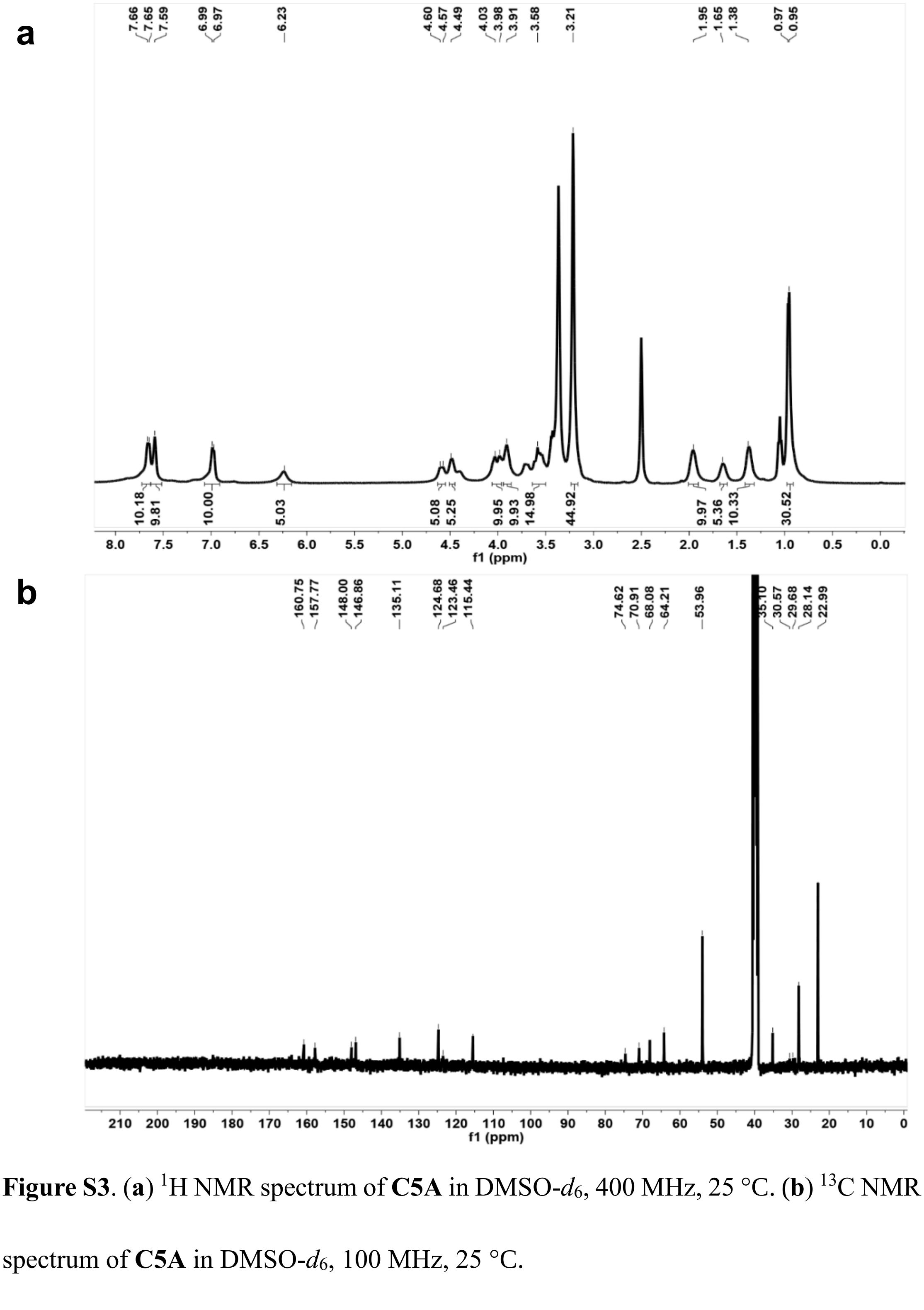

Figure S3. (**a**) ^{1}H NMR spectrum of **C5A** in DMSO-d_6, 400 MHz, 25 °C. (**b**) ^{13}C NMR spectrum of **C5A** in DMSO-d_6, 100 MHz, 25 °C.

3 Supporting Figures

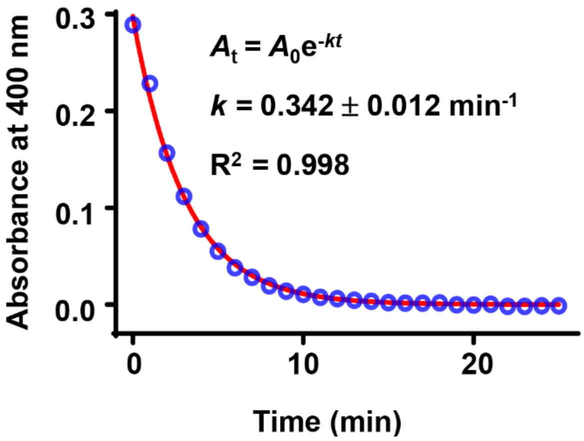

Figure S4. Absorbance of C5A (10 μM) at 400 nm as a function of time following addition of SDT (2 mM) in PBS buffer (10 mM), and the corresponding fitting curve according to quasi-first order reaction decay model. Experimental conditions: pH 7.4, 25 °C. Each experiment was performed in triplicate.

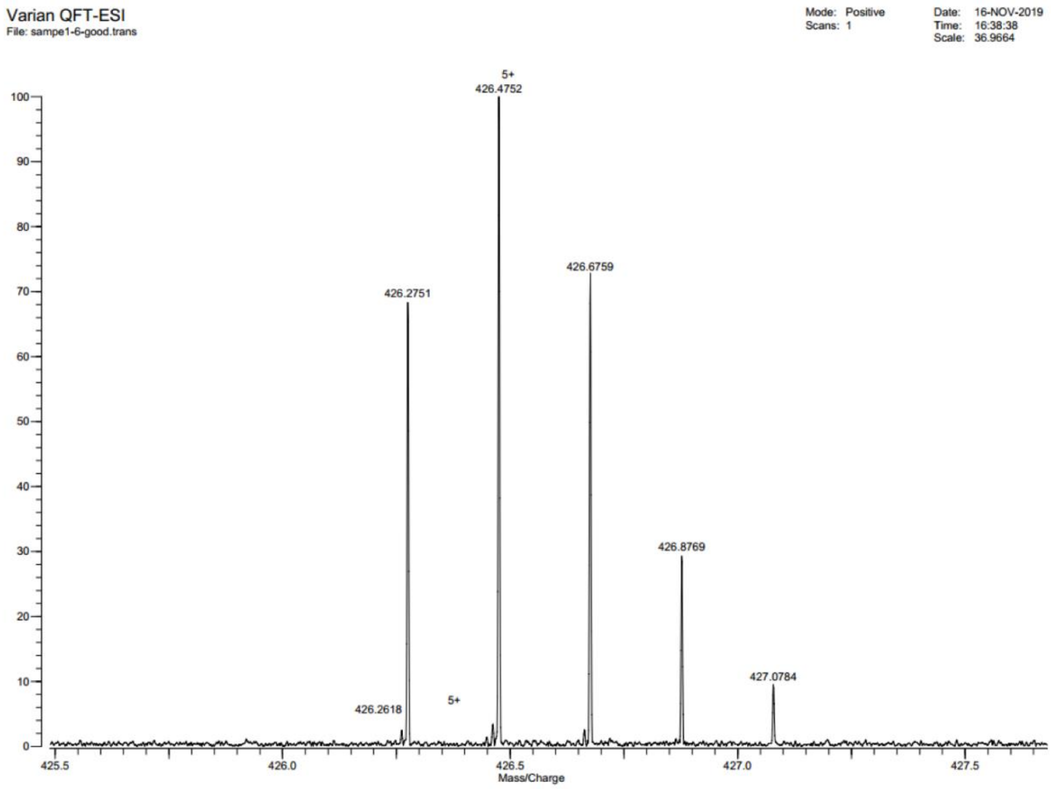

Figure S5. Mass spectrum of **C5A** (QFT-ESI).

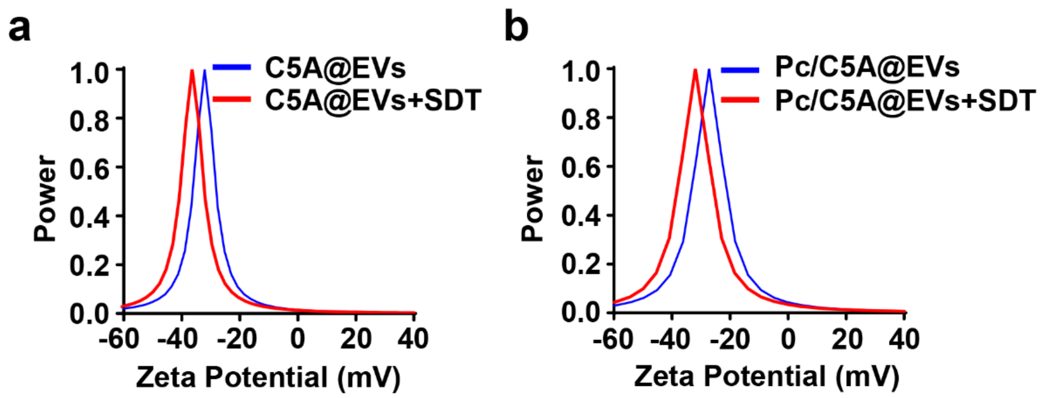

Figure S6. Zeta potential detection of C5A@EVs and Pc/C5A@EVs upon incubation with or without SDT in PBS buffer. (**a**) Zeta potential measure of C5A@EVs in the absence (blue) or presence of SDT (red) in PBS buffer (10 mM, pH = 7.4) at 25 ℃. (**b**) Zeta potential measure of Pc/C5A@EVs in the absence (blue) or presence of SDT (red) in PBS buffer (10 mM, pH = 7.4) at 25 ℃. Each experiment was performed in triplicate.

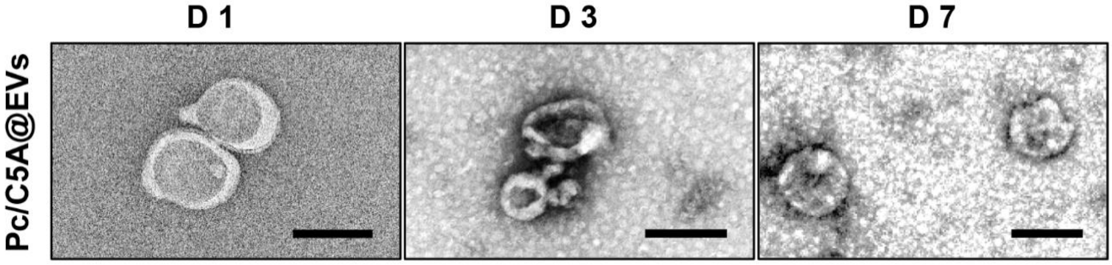

Figure S7. Representative TEM images of Pc/C5A@EVs on day 1, 3 and 7. Scale bar, 100 nm.

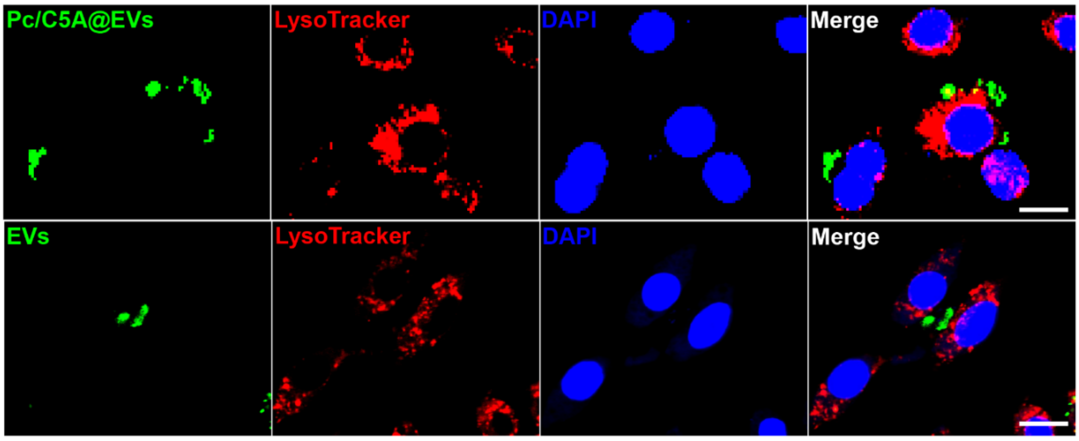

Figure S8. *In vitro* colocalization of Pc/C5A@EVs and MSC-EVs with LysoTracker Red in TECs by CLSM. Green: Pc/C5A@EVs or MSC-EVs; Red: LysoTracker; Blue: DAPI. Scale bar, 10 μm.

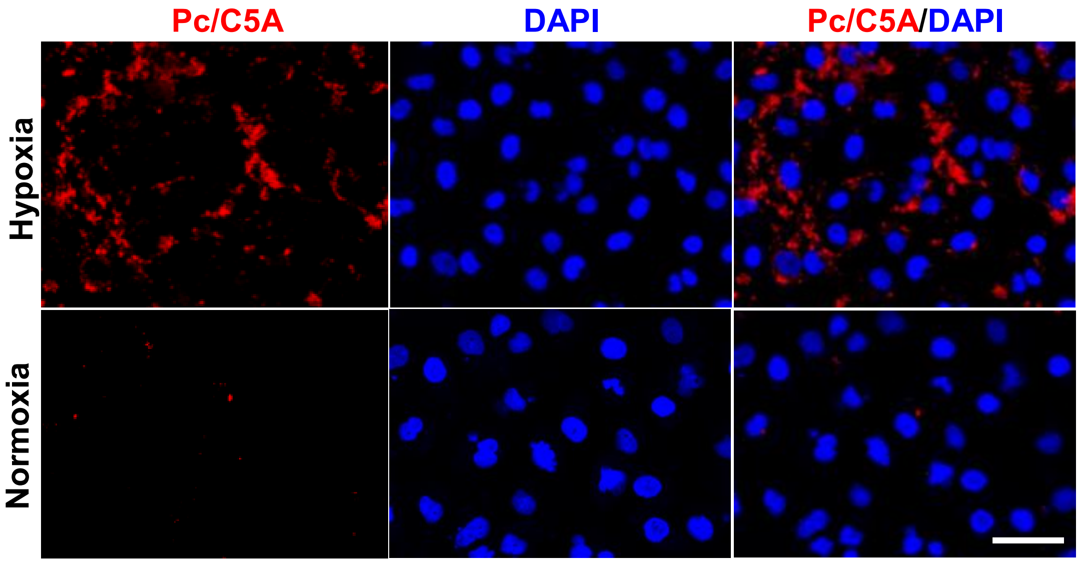

Figure S9. CLSM images of Pc/C5A under hypoxic or normoxic condition. Scale bar, 40 μm.

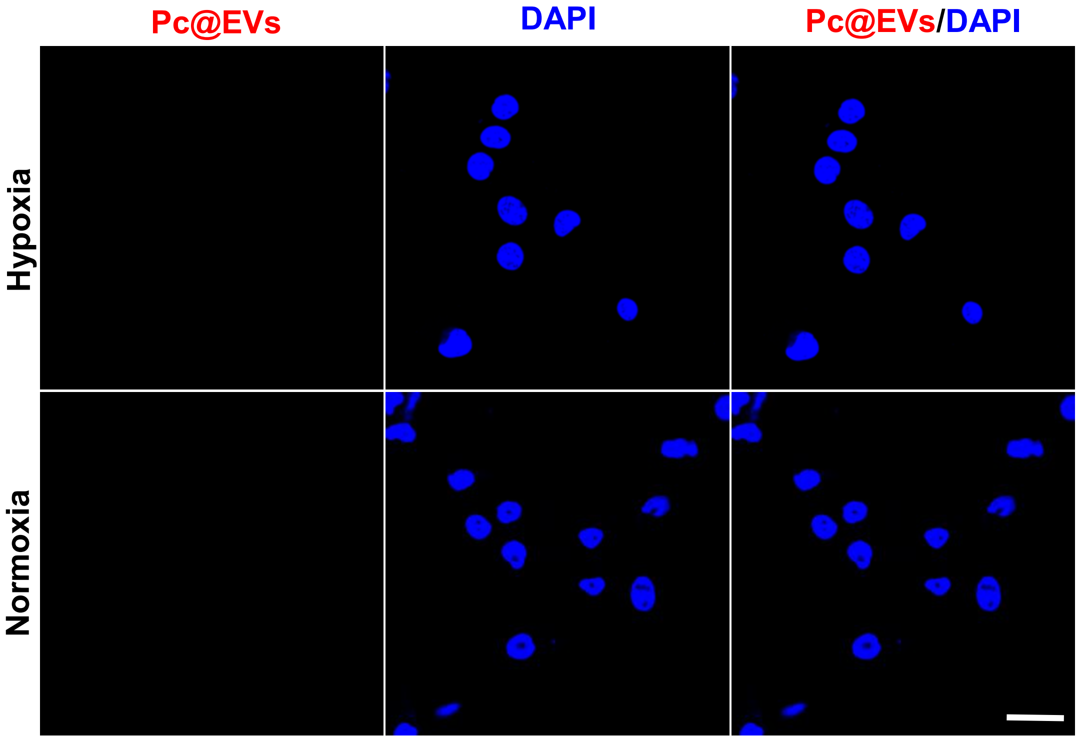

Figure S10. CLSM images of Pc@EVs under hypoxic or normoxic condition. Scale bar, 50 μm.

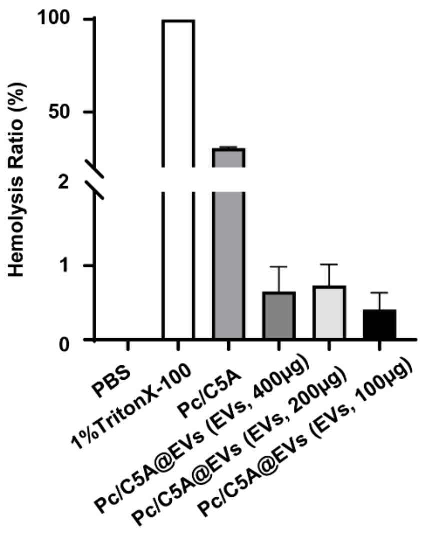

Figure S11. Hemolysis rate of Pc/C5A and Pc/C5A@EVs after incubation with mouse erythrocytes. Each experiment was performed in triplicate.

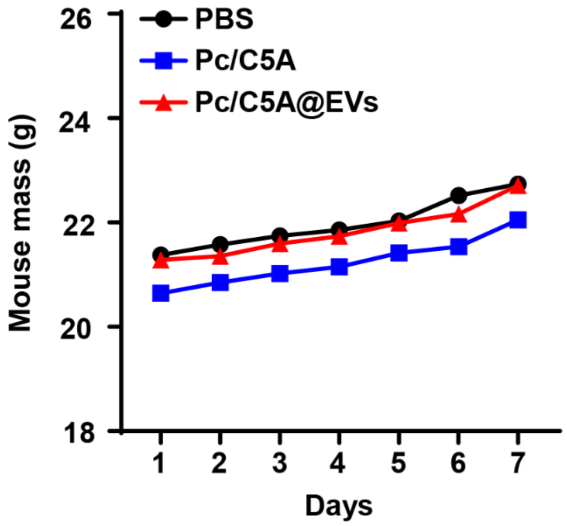

Figure S12. Body weight changes of the mice with different treatments (*n* = 5).

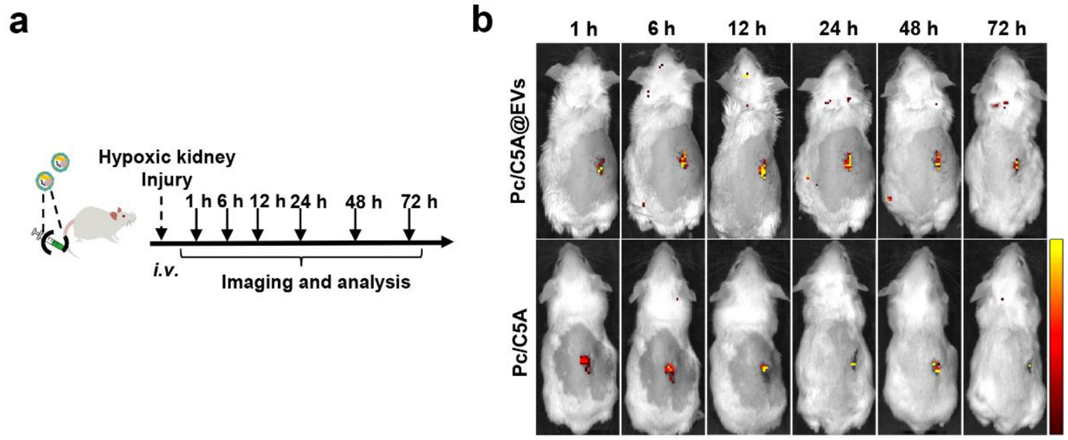

Figure S13. *In vivo* imaging of kidney hypoxia via Pc/C5A@EVs and Pc/C5A. (**a**) Illustration of the imaging tracing strategy after intravenous injection of Pc/C5A@EVs or Pc/C5A in unilateral hypoxic renal injury mice. (**b**) *In vivo* fluorescence images of Pc/C5A@EVs and Pc/C5A in unilateral hypoxic renal injury mice.

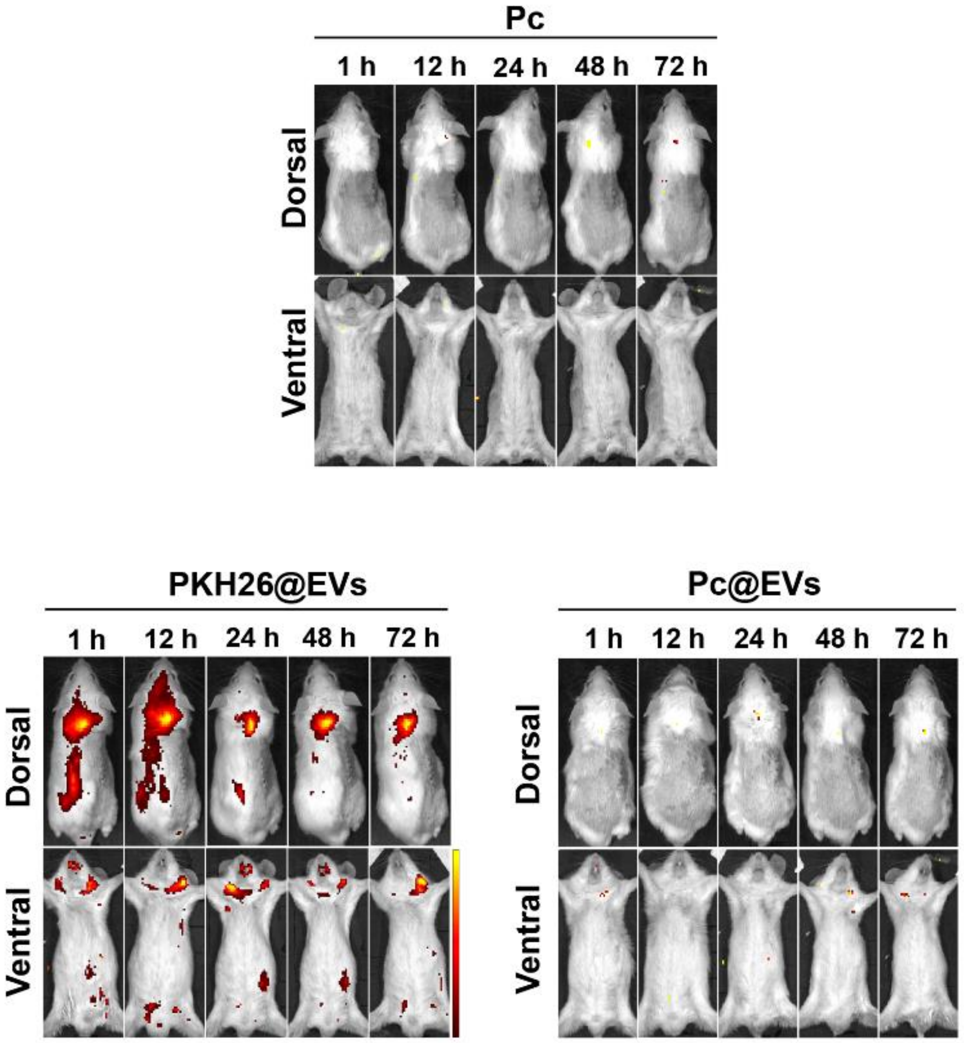

Figure S14 *In vivo* imaging of hypoxia-injured mice with intravenous injection of Pc, Pc@EVs or PKH26@EVs at different time intervals (*n* = 3).

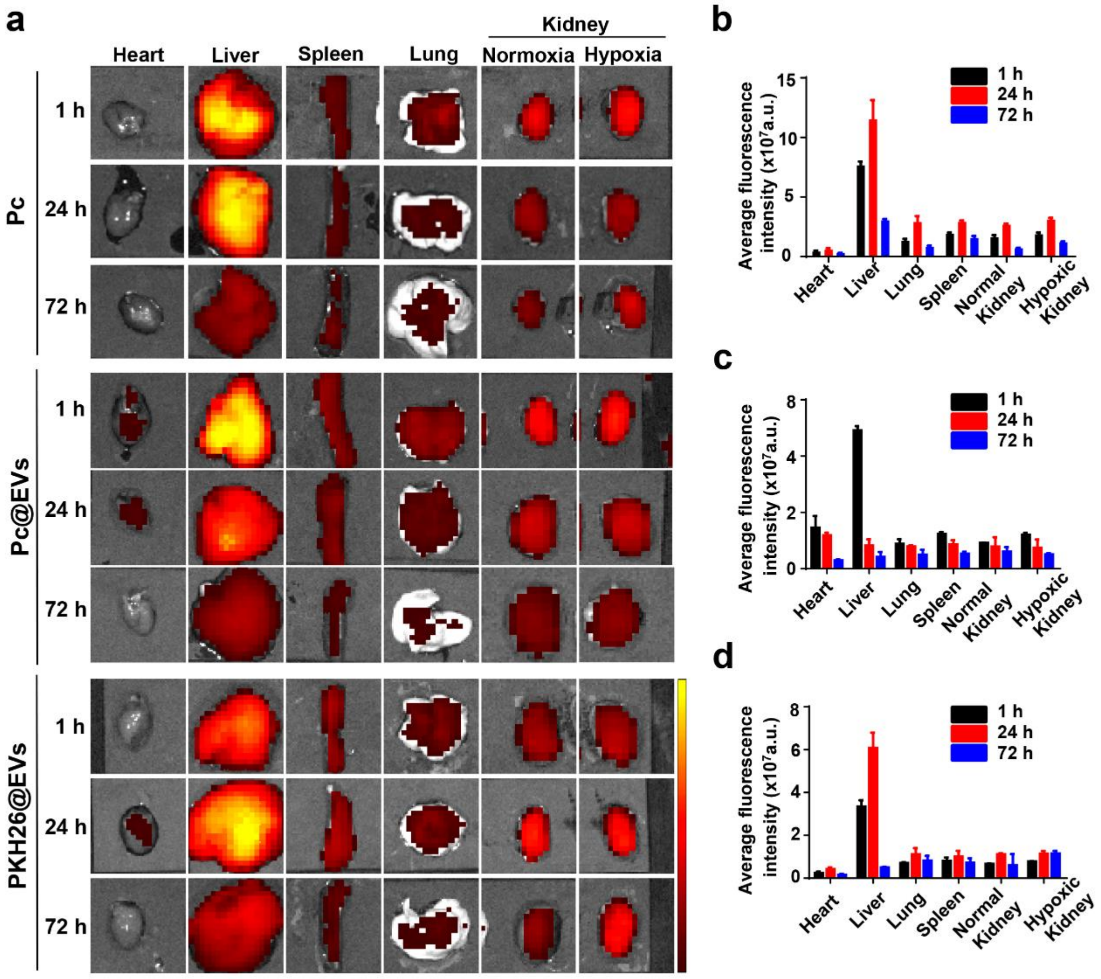

Figure S15. (a) *Ex vivo* images of major organs at the designed time points after Pc, Pc@EV or PKH26@EV administration. (**b-d**) Time-dependent fluorescence intensity changes in major organs at designated time intervals after sacrificing renal hypoxia-injured mice (n = 5).

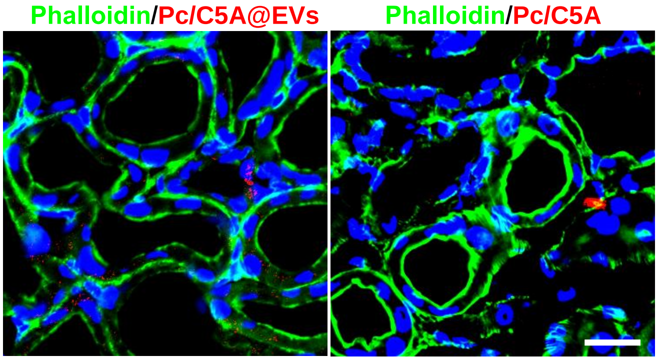

Figure S16. Representative CLSM images of kidney slices from the renal hypoxia-injured mice after administration of Pc/C5A@EVs or Pc/C5A for 24 h. Phalloidin: green; Pc/C5A, Pc/C5A@EVs: red; DAPI: blue. Scale bars = 20 μm.

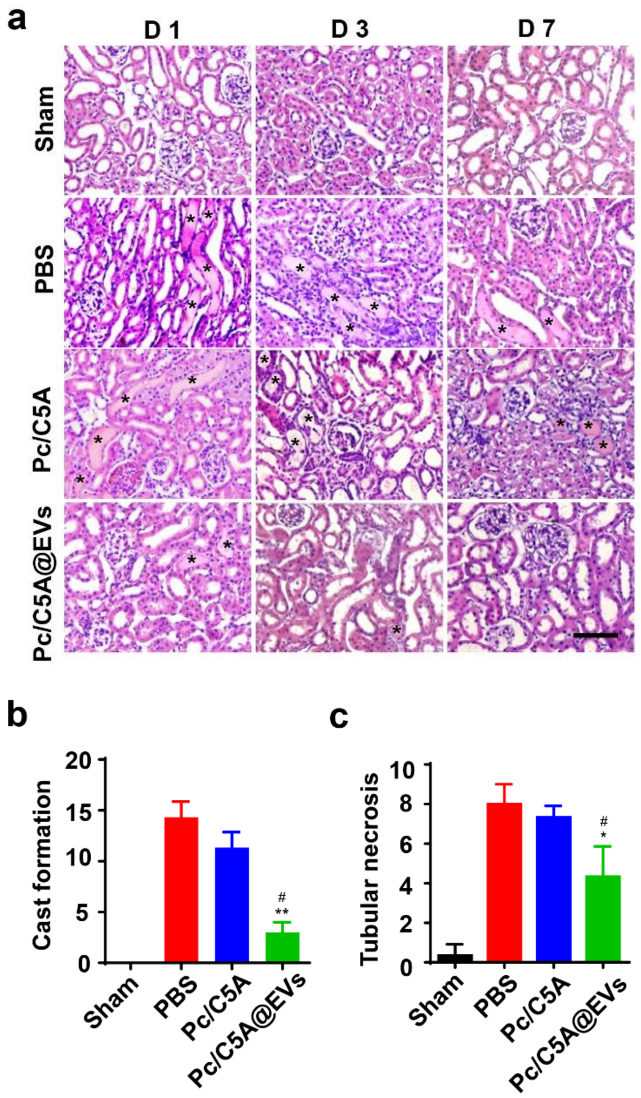

Figure S17. (**a**) Representative H&E-staining images of kidney sections on day 1, 3 and 7 after injection. Massive necrosis in the proximal tubules with hyaline cast formation (asterisks) was observed, and administration of Pc/C5A@EVs largely prevented histopathologic alterations after hypoxic injury. Scale bar, 50 μm. (**b-c**) Quantitative histological assessment of hyaline cast formation (B) and tubular necrosis (**c**) on day 3 postinjection ($n = 5$; * $P < 0.05$ compared with PBS; ** $P < 0.01$ compared with PBS; $^{\#}$ $P < 0.05$ compared with Pc/C5A). Each experiment was performed in triplicate.

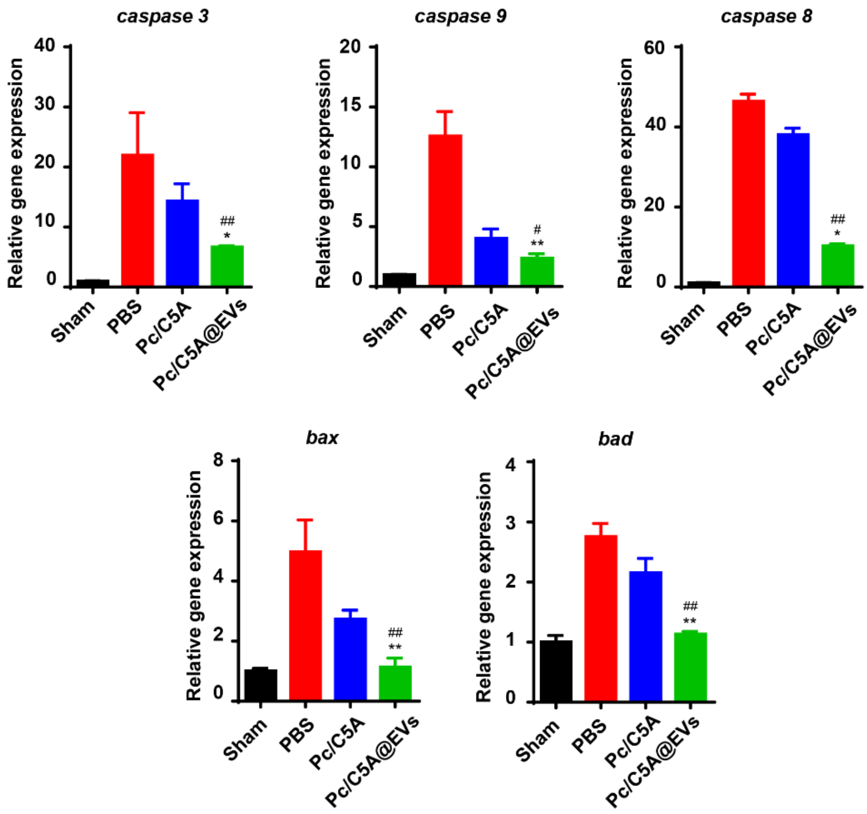

Figure S18. RT-qPCR analysis of the expression of apoptosis-related genes on day 7 postinjection (n = 5; * P < 0.05 compared with PBS; ** P < 0.01 compared with PBS; $^{\#}$ P < 0.05 compared with Pc/C5A, $^{\#\#}$ P < 0.01 compared with Pc/C5A). Each experiment was performed in triplicate.

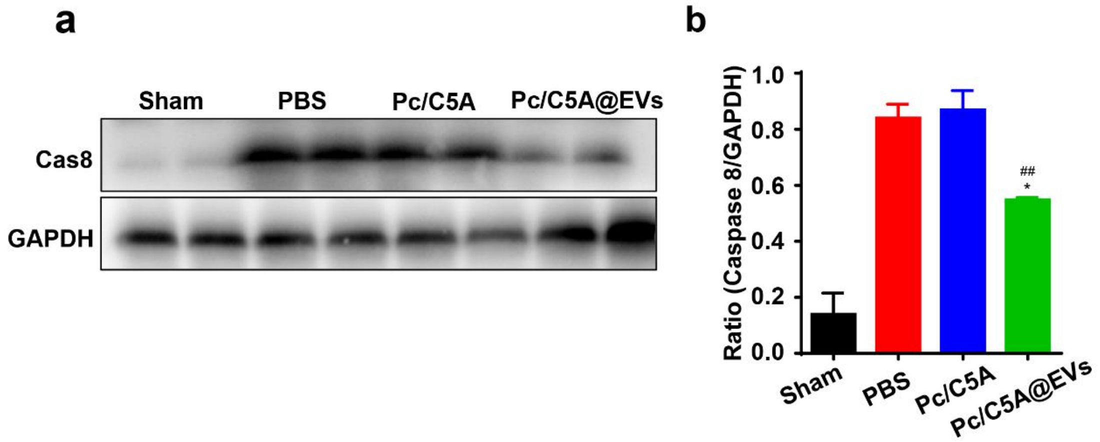

Figure S19. Representative Western blot showing Caspase 8 expression in kidney tissues with different treatments on day 7 postinjection. (**a**) Representative Western blot images of Caspase 8 in different groups. (**b**) Quantitative analysis of Western blot (n = 3; * $P < 0.05$ compared with PBS; $^{\#\#}$ $P < 0.01$ compared with Pc/C5A). Each experiment was performed in triplicate.

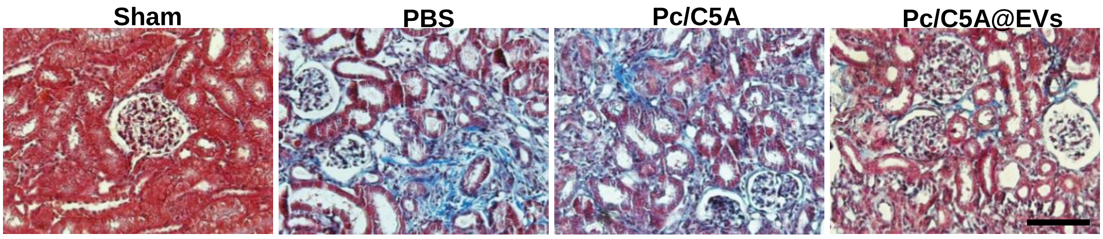

Figure S20. Representative images of Masson trichrome staining in different groups (*n* = 8). Scale bars, 50 μm.

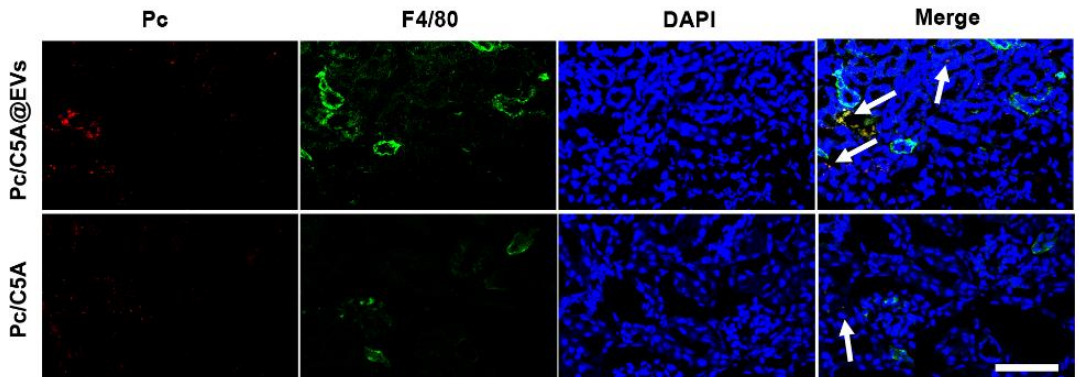

Figure S21. Representative images of immunofluorescence staining of F4/80 (green) and Pc/C5A@EVs or Pc/C5A (red) in the kidney tissues in different groups. DAPI: blue. Scale bar = 100 μm.

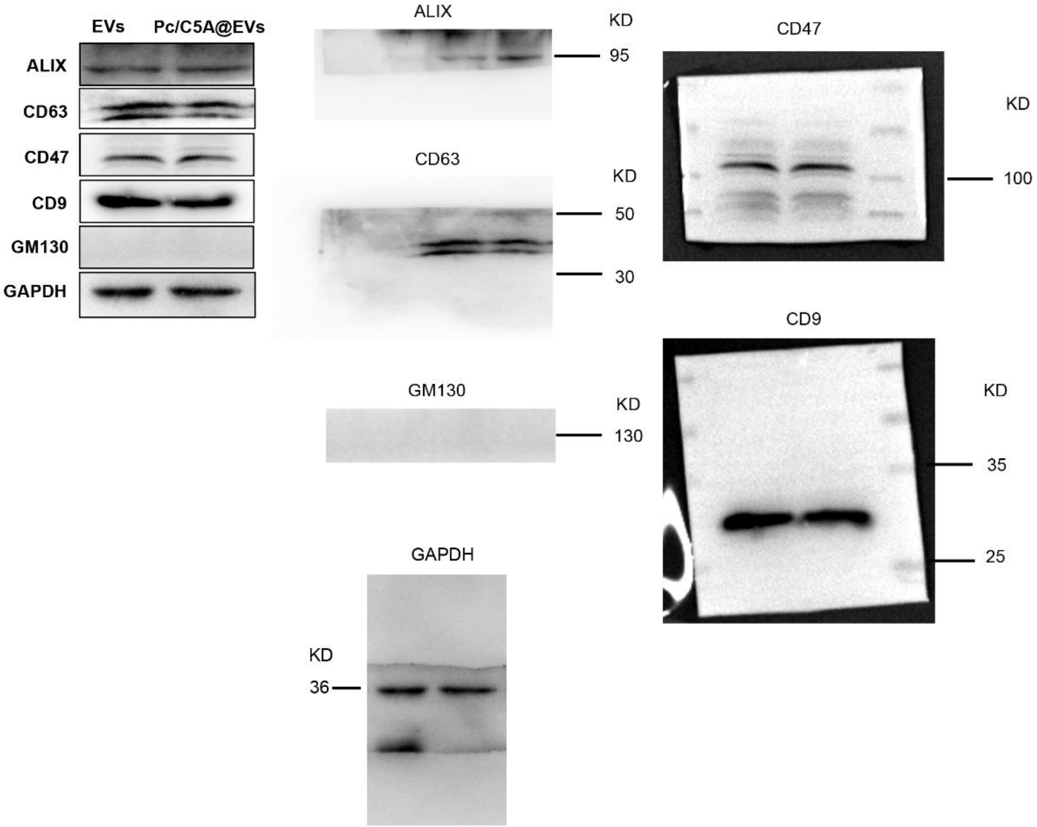

Figure S22. The raw data for Western blot in Figure 2d.

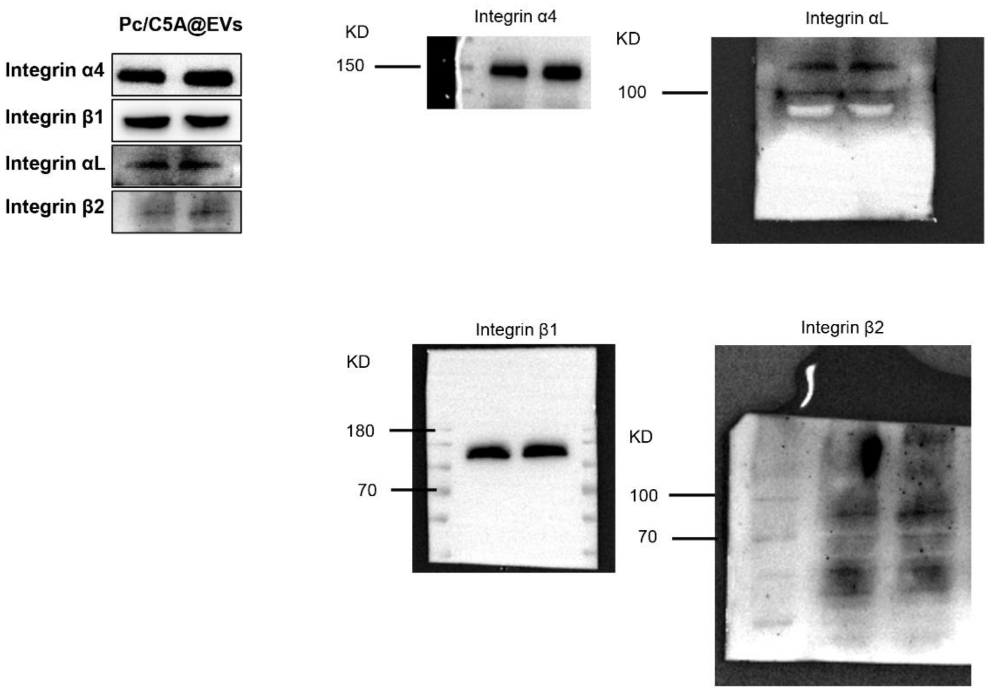

Figure S23. The raw data for Western blot in Figure 6a.

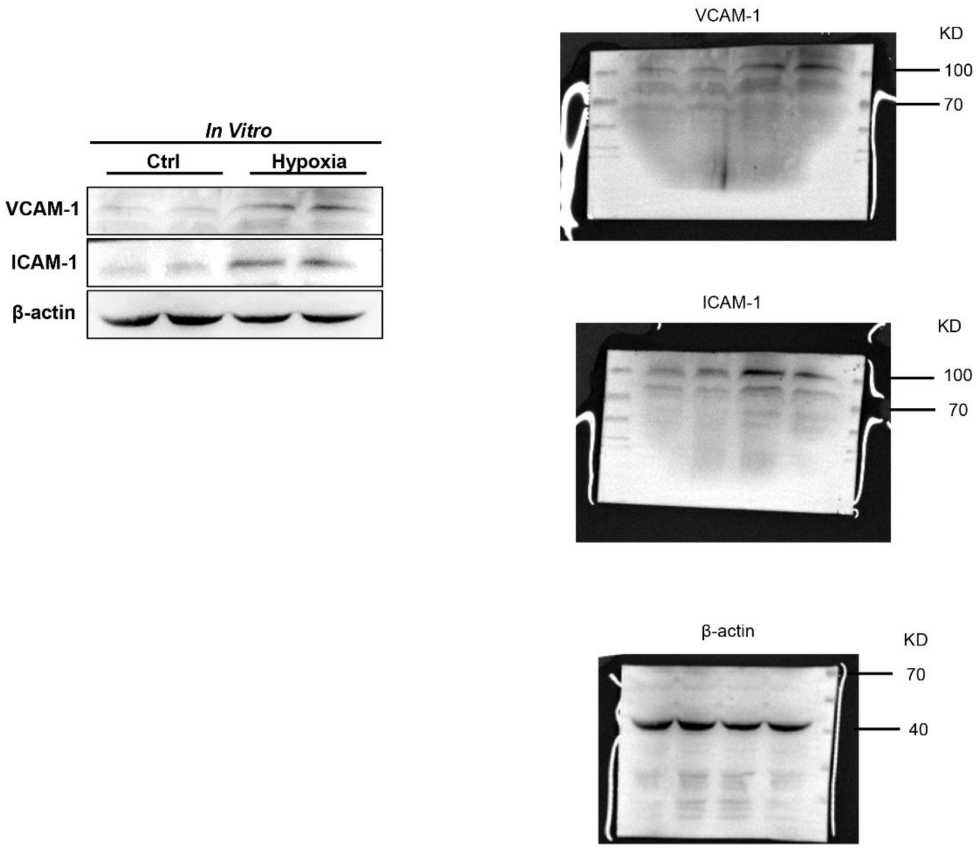

Figure S24. The raw data for Western blot in Figure 6b.

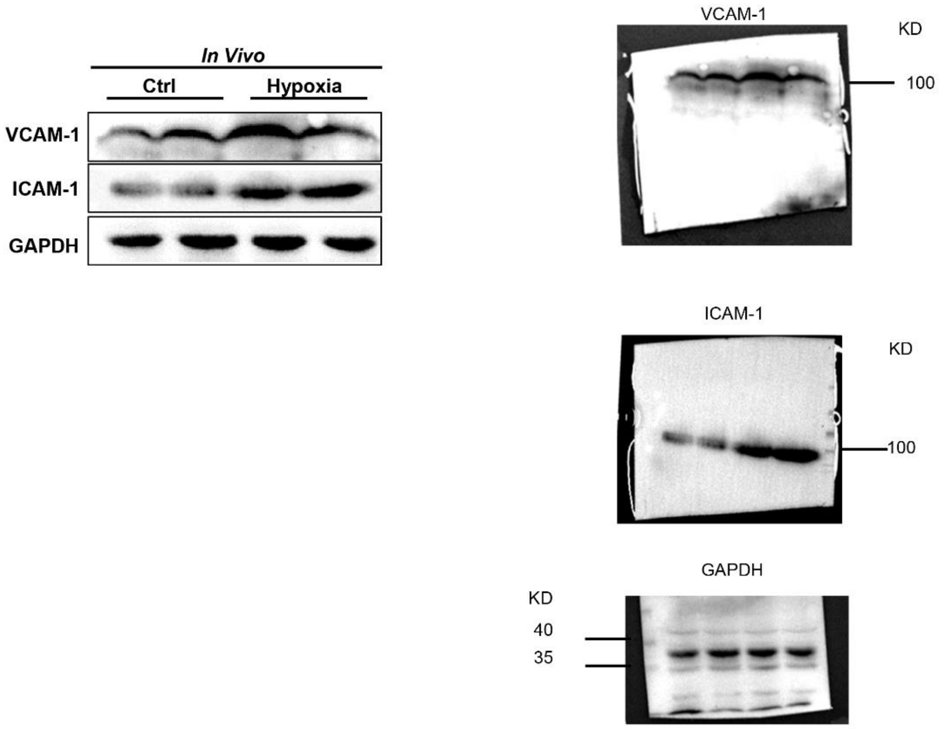

Figure S25. The raw data for Western blot in Figure 6c.

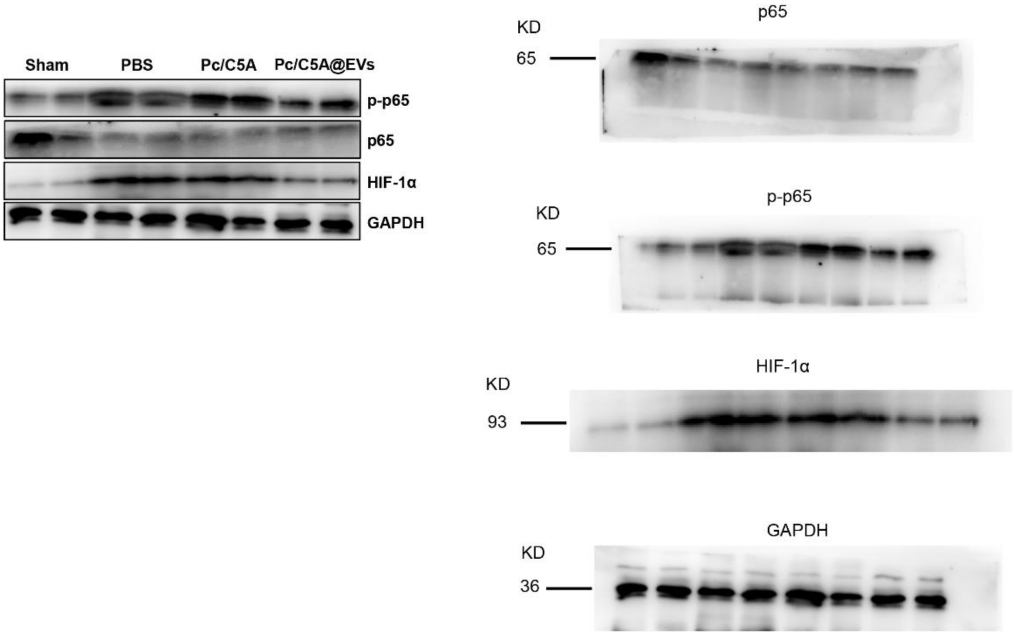

Figure S26. The raw data for Western blot in Figure 7j.

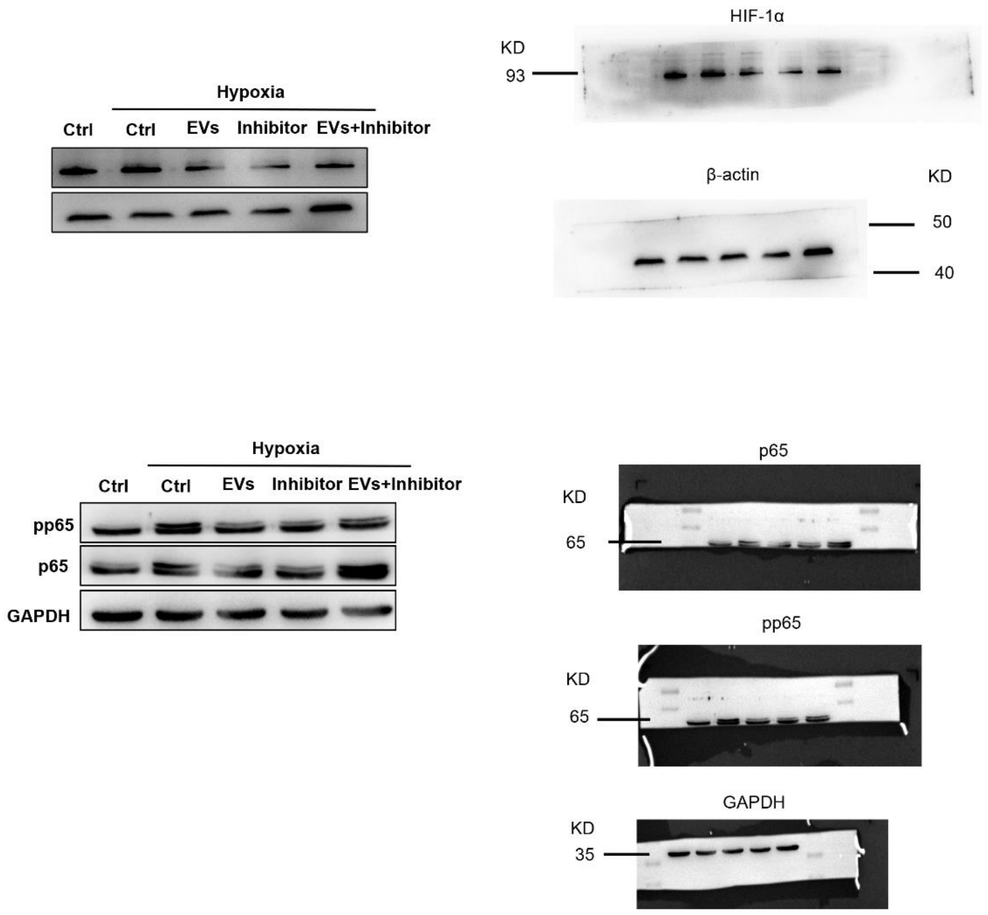

Figure S27. The raw data for Western blot in Figure 8k-l.

Table S1 Primers used in the RT-qPCR assay

Transcription		Primer sequences
HIF-1α	Forward primer	AGATTCTGTTTGTTGAAGGGAG
	Reverse primer	AGGTGGATATGTCTGGGTTGA
GAPDH	Forward primer	GCATGGCCTTCCGTGTTC
	Reverse primer	GATGTCATCATACTTGGCAGGTTT
TNF-α	Forward primer	CATCTTCTCAAAATTCGAGTGACAA
	Reverse primer	TGGGAGTAGACAAGGTACAACCC
IL-1β	Forward primer	TGCCACCTTTTGACAGTGATG
	Reverse primer	AAGGTCCACGGGAAAGACAC
MCP-1	Forward primer	ACTGAAGCCAGCTCTCTCTTCCTC
	Reverse primer	TTCCTTCTTGGGGTCAGCACAGAC
IL-10	Forward primer	GCTCTTACTGACTGGCATGAG
	Reverse primer	CGCAGCTCTAGGAGCATGTG
CASP3	Forward primer	TGAAGGGGTCATTTATGGGACA
	Reverse primer	CCAGTCAGACTCCGGCAGTA
CASP8	Forward primer	TGCTTGGACTACATCCCACAC
	Reverse primer	GTTGCAGTCTAGGAAGTTGACC
CASP9	Forward primer	GGCTGTTAAACCCCTAGACCA
	Reverse primer	TGACGGGTCCAGCTTCACTA
BAX	Forward primer	TGAAGACAGGGGCCTTTTTG
	Reverse primer	AATTCGCCGGAGACACTCG
BAD	Forward primer	AAGTCCGATCCCGGAATCC
	Reverse primer	GCTCACTCGGCTCAAACTCT

4 REFERENCES FOR SUPPORT MATERIAS

1. Agnoletti M, Rodriguez-Rodriguez C, Klodzinska SN, Esposito TVF, Saatchi K, Morck Nielsen H, Häfeli UO. Monosized Polymeric Microspheres Designed for Passive Lung Targeting: Biodistribution and Pharmacokinetics after Intravenous Administration. ACS Nano. 2020; 14:6693-6706.
2. Zhuang M, Du D, Pu L, Song H, Deng M, Long Q, Yin X, Wang Y, Rao L. SPION-Decorated Exosome Delivered BAY55-9837 Targeting the Pancreas through Magnetism to Improve the Blood GLC Response. Small. 2019; 15:e1903135.
3. Cao H, Yue Z, Gao H, Chen C, Cui K, Zhang K, Cheng Y, Shao G, Kong D, Li Z, et al. In Vivo Real-Time Imaging of Extracellular Vesicles in Liver Regeneration via Aggregation-Induced Emission Luminogens. ACS Nano. 2019; 13:3522-3533.
4. Zheng Z, Geng W-C, Gao J, Wang Y-Y, Sun H, Guo D-S. Ultrasensitive and specific fluorescence detection of a cancer biomarker via nanomolar binding to a guanidinium-modified calixarene. Chem. Sci. 2018; 9:2087-2091.
